# Multi-parametric profiling of IL-7-augmented GD2.CART products in a phase 1 clinical trial

**DOI:** 10.1016/j.isci.2025.113680

**Published:** 2025-10-06

**Authors:** Sarah Schulenberg, Martí Farrera-Sal, Michelle Loeser, Lukas Ehlen, Stephan Schlickeiser, Samira Picht, Lena Peter, Marco Mai, Candise L. Tat, Jacqueline Keye, Desiree Kunkel, Frank Lin, Cliona M. Rooney, Bilal Omer, Michael Schmueck-Henneresse

**Affiliations:** 1 Berlin Institute of Health (BIH) at Charité – Universitätsmedizin Berlin, BIH Center for Regenerative Therapies (BCRT), Experimental Immunotherapy, Augustenburger Platz 1, 13353 Berlin, Germany; 2 Charité - Universitätsmedizin Berlin, Corporate Member of Freie Universität Berlin, Humboldt-Universität zu Berlin and Berlin Institute of Health, Department of Anesthesiology and Intensive Care Medicine, Berlin, Germany; 3 CheckImmune GmbH, BerlinBioCube, (Haus D95), Robert-Rössle-Str. 10, 13125 Berlin, Germany; 4 Center for Cell and Gene Therapy, Texas Children’s Hospital, Houston Methodist Hospital, Baylor College of Medicine, Houston, TX, US; 5 Texas Children’s Cancer and Hematology Centers, Texas Children’s Hospital, Baylor College of Medicine, Houston, TX, US; 6 Berlin Institute of Health at Charité - Universitätsmedizin Berlin, Flow & Mass Cytometry Core Facility, Augustenburger Platz 1, 13353 Berlin, Germany

**Keywords:** Health sciences, Immunology, Laboratory medicine, Medicine, Oncology

## Abstract

CAR T cell (CART) therapy holds promise for cancer treatment, but heterogeneity among products limits clinical effectiveness, making systematic profiling essential to identify predictors of success. Recently, a phase 1 clinical trial investigated whether a constitutively active IL-7 receptor (C7R) could safely improve the function and persistence of GD2-directed CARTs (GD2.CARTs) in pediatric patients with high-grade CNS tumors. We analyzed infusion products from trial participants using a custom-designed 33-color full spectrum flow cytometry (FSFC) panel combined with an image-based tumor killing assay to characterize CART products and evaluate the impact of C7R on GD2.CART performance. Patient-specific variations in T cell composition were linked to therapeutic success, with C7R co-expression enhancing the functional phenotype of GD2.CARTs compared to CAR-only products. Unsupervised clustering identified CD8^+^ T cells associated with clinical responses, marked by activation, infiltration, resilience, and cytotoxicity. Our FSFC-based profiling approach reveals determinants of CART efficacy and supports strategies to optimize adoptive immunotherapy.

## Highlights

• 33-marker spectral cytometry enables high-dimensional CART product profiling
• C7R co-expression enhances GD2.CART activation, cytotoxicity, and infiltration
• Distinct CD8^+^ T cell clusters are enriched in products linked to clinical response
• Spectral cytometry-based profiling approach reveals determinants of CART efficacy

## Graphical abstract

**Figure:**
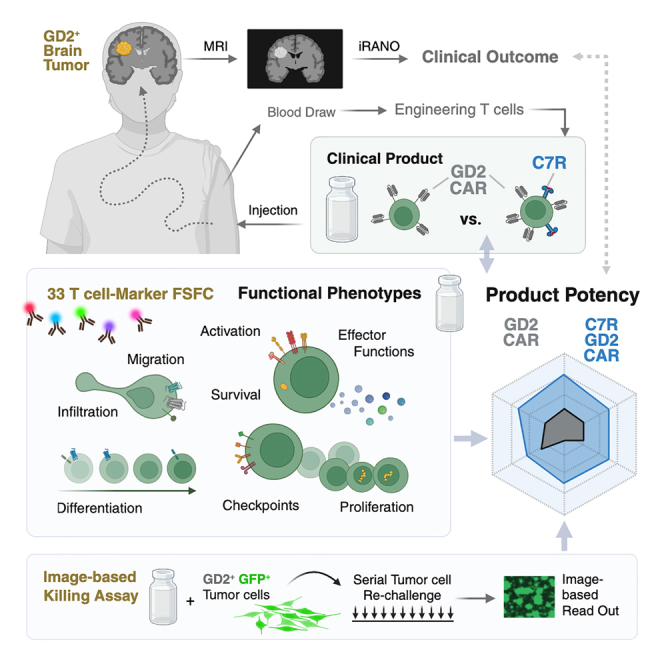

## Introduction

Chimeric antigen receptor T cell (CART) therapy has transformed treatment paradigms for hematologic B cell malignancies but has limited efficacy against other tumors.^1^ GD2-directed CARTs (GD2.CARTs) show promise for treating diffuse midline glioma (DMG) and other GD2-expressing brain tumors, though their efficacy is challenged by the immunosuppressive tumor microenvironment (TME) and poor T cell persistence. To address this, a constitutively active IL-7 receptor (C7R) was implemented that enhances CART survival, metabolic fitness, and antitumor activity by activating IL-7-independent STAT5 signaling and overcomes cytokine deprivation at tumor sites without affecting bystander immune cells.^2^ Building on preclinical studies that demonstrated enhanced CART proliferation, persistence, and tumor-killing capacity by the C7R, even in cytokine-depleted environments, a phase 1 clinical trial was initiated to assess the safety and efficacy of C7R-modified GD2.CARTs after lymphodepleting chemotherapy in children with recurrent GD2-expressing CNS tumors.^3^ In total, 11 patients (ages 4–18) were treated without dose-limiting toxicity. The initial cohort (*n* = 3) received GD2.CARTs alone and experienced disease progression (PD) after brief improvement of residual neurologic deficits (≤3 weeks), whereas eight patients treated with C7R-GD2.CARTs exhibited temporary improvement in baseline neurologic deficits (range, 2 to >12 months), and 7 of 8 (88%) remained eligible for additional treatment cycles. Notably, within our analyzed cohort of 10 products, one of the DMG patients demonstrated a partial radiographic response after 6 weeks of treatment, alongside clinical improvement. These findings suggest that the addition of C7R can safely enhance GD2.CART function, but variability in patient outcomes raises important questions about the influence of T cell characteristics on therapeutic success. Understanding the effect of CART product heterogeneity on clinical efficacy is crucial as differences in T cell composition and functional properties, such as memory differentiation, activation, and resistance to exhaustion, significantly impact therapeutic efficacy and long-term protection.^4^^,^^5^^,^^6^^,^^7^ In solid tumors, the ability of T cells to migrate to tumor sites and establish localized immune responses, particularly through tissue-resident memory T cells, is important for sustained anti-tumor activity.^8^ However, the suppressive TME often induces exhaustion in T cells, characterized by the upregulation of checkpoint receptors, limiting their functionality, and reducing their therapeutic potential.^9^

Full spectrum flow cytometry (FSFC) enables high-dimensional, rapid characterization of CART products by allowing simultaneous detection of multiple T cell parameters. While published FSFC panels often focus on T cells within heterogeneous populations, such as human peripheral blood mononuclear cells (PBMCs), profiling CART products requires detection of numerous functionally relevant markers within a single cell type, ideally without increasing methodological complexity. We designed a 33-color FSFC panel to analyze T cell characteristics in patient-derived clinical CART products, and applied it to a cohort of ten patients in a phase 1 clinical trial that assessed the safety and efficacy of C7R-modified GD2.CARTs in children with recurrent GD2-expressing CNS tumors, serving as a model study to validate panel functionality. Our approach allowed a comparison of the CART product compositions identifying critical T cell features within the cell products. An in-depth assessment of T cell attributes included memory differentiation, activation states, exhaustion markers, cytokine production, and cytotoxic potential, providing a functional landscape of CART products. We integrated FSFC-derived phenotypic and functional data with image-based recording of serial tumor-cell killing to assess dynamic functional properties of the CART products, associating these findings with therapeutic efficacy based on tumor progression following treatment with GD2.CARTs with and without C7R.^3^ Variability in T cell composition was observed with favorable T cell-features in CARTs expressing C7R compared to GD2.CART-only products. Within the cell products associated with the best clinical response, distinct CD8^+^ T cell clusters displaying markers related to T cell activation, cytotoxicity, resilience, and tissue infiltration potential were enriched. By integrating high-dimensional flow cytometry with an assessment of serial tumor cell-killing, we provide a framework for characterizing CART product composition, linking functional heterogeneity to patient-specific responses and informing strategies to optimize adoptive immunotherapy in solid tumors.

## Results

### Evaluation of T cell characteristics in CART therapy using a 33-marker flow cytometry panel

**Figure 1:**
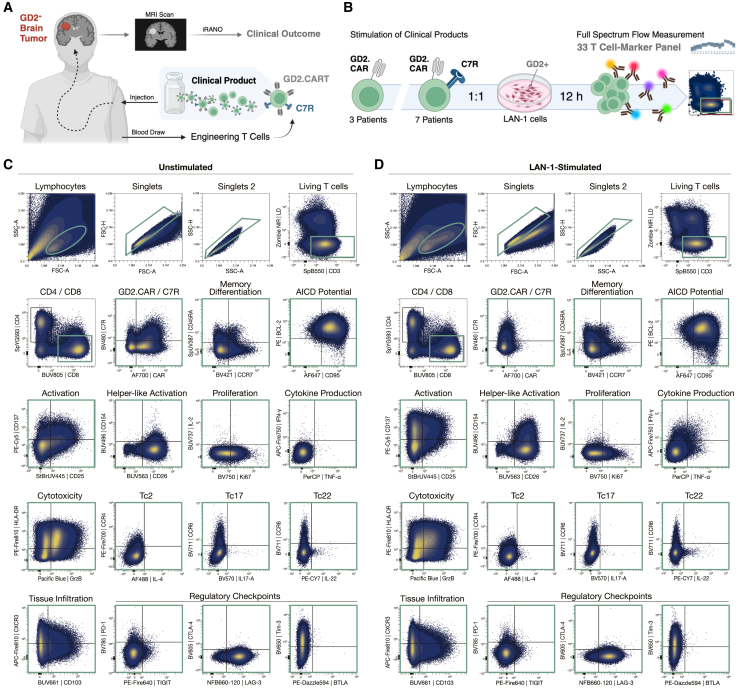
Gating strategy and validation of a 33-marker FSFC panel for functional profiling of clinical CART products (A) Schematic illustration of the phase 1 clinical study assessing efficacy and safety of autologous CART products targeting GD2-positive brain tumors. Clinical outcome was evaluated by immunotherapy response assessment for neuro-oncology (iRANO) after 6 weeks, with tumor mass classified via MRI scans. Created with BioRender.com (B) For analysis, T cell products harboring the GD2.CAR alone (*n* = 3) or in combination with the C7R (*n* = 7) were stimulated with GD2-expressing LAN-1 tumor cells at a 1:1 ratio for 12 h. Samples were stained with a 33-color panel to detect T cell markers using full spectrum flow cytometry. Created with BioRender.com In (C) unstimulated and (D) stimulated T cell products, single events were identified based on size and granularity (FSC, SSC), followed by doublet exclusion using height versus area (FSC, SSC). Viable CD4^−^CD8^+^ T cells are shown, with the expression of markers relevant for memory differentiation (CCR7 and CD45RA), apoptosis regulation (CD95 and BCL-2), activation (CD25, CD137, CD26, and CD154), proliferation (Ki67 and IL-2), cytokine production (TNF-α and IFN-γ), cytotoxicity (GrzB and HLA-DR), T cell subsets (CCR4, CCR6, IL-4, IL-17, and IL-22), tissue infiltration (CD103 and CXCR3), and exhaustion-related checkpoint expression (TIGIT, LAG-3, BTLA, PD-1, CTLA-4, and Tim-3) displayed as a combined overlay of the T cell products analyzed (*n* = 10). Subsequent gates are shown in green.

We developed a unique 33-marker FSFC panel to assess the immunophenotypic and functional characteristics of clinical GD2.CARTs from the phase 1 trial for pediatric GD2-positive brain tumors.^3^ In this trial, patients received autologous second-generation GD2.CARTs featuring a 4-1BB-derived endodomain with or without the C7R (Figure 1A). Patients co-expressing the C7R experienced neurological improvement for a median of 6 months.^2^ Tumor response was assessed after 6 weeks of treatment, categorizing patients as partial response (PR), stable disease (SD), or PD based on radiographic findings. These response categories were used to evaluate the association between CART characteristics and treatment, and the impact of the C7R modification on tumor progression. Our 33-color FSFC panel assessed key T cell characteristics, including subset (CD3, CD4, and CD8), memory differentiation (CD45RA, CCR7), tissue infiltration (CD103), homing (CXCR3), activation (CD137, CD154, CD25, CD26, HLA-DR, IFN-γ, TNF-α), proliferation (Ki67), survival (BCL-2), activation-induced cell death potential (CD95), checkpoint expression (BTLA, CTLA-4, LAG-3, PD-1, TIM-3, and TIGIT), cytokine production (IL-2, IL-4, IL-17A, and IL-22), chemokine receptor expression (CCR4, CCR6), CAR expression (14g2a ScFv), and C7R presence (CD34 ectodomain) (Tables 1 and 2). Marker selection was driven by relevant biological questions (Table S1). While the panel included 33 markers, we designed the fluorophore combinations to maintain low overall complexity, as measured by a complexity index of 22.7. This index quantitatively reflects the diversity and overlap of fluorescence signals in the sample, ensuring optimal resolution without excessive signal interference (Figure S1).

**Table 1:** Marker used for extracellular flow cytometry staining

Marker	Clone	Fluorochrome	Vendor
CD45RA	HI100	SUV387	Biolegend
CD25	MEM-181	BUV445	BioRad
CD26	M-A261	BUV563	BD
CD103	Ber-ACT8	BUV661	BD
CD4	SK3	SYG593	Biolegend
CD8	RPA-T8	BUV805	BD
CCR7	G043H7	BV421	Biolegend
14g2a	1A7	AF700	Abcam (Lightning-Link Conjugation Kit)
CD34	563	BV480	BD
CTLA-4	BNI3	BV605	Biolegend
TIM-3	F38-2E2	BV650	Biolegend
CCR6	G034E3	BV711	Biolegend
PD-1	EH12.2H7	BV785	Biolegend
CD3	SK7	SB550	Biolegend
BTLA	MIH26	PE/Dazzle	Biolegend
TIGIT	A15153G	PE/Fire640	Biolegend
CCR4	L291H4	PE/Fire700	Biolegend
HLA-DR	L243	PE/Fire810	Biolegend
CD95	DX2	AF647	Biolegend
CXCR3	G025H7	APC/Fire810	Biolegend

**Table 2:** Marker used for intracellular flow cytometry staining

Marker	Clone	Fluorochrome	Vendor
IL-4	MP4-25D2	AF488	Biolegend
IFN-γ	4S.B3	APC/Fire750	Biolegend
CD154	24–31	BUV496	BD
IL-2	MQ1-17H12	BUV737	BD
IL17-A	BL168	BV570	Biolegend
KI67	ki-67	BV750	Biolegend
LAG-3	3DS223H	NFB660	Thermo
GrzB	QA16A02	PacBlue	Biolegend
BCL-2	100	PE	Biolegend
CD137	4B4-1	PE/Cy5	Biolegend
IL-22	MH22B2	PE/Cy7	BD
TNF-α	MAb11	PerCP	Biolegend

FSFC panel establishment.

We validated the panel using healthy human PBMCs and expanded T cells following established protocols,^10^ determining optimal antibody dilutions (Figure S2), and reference controls (Table S2; Figure S3). To evaluate functionality of the clinical CART products, we stimulated them with GD2-expressing LAN-1 neuroblastoma cells at a 1:1 ratio and analyzed the expression of 33 markers via FSFC 12 h post-stimulation. We investigated ten clinical products of which three contained only the GD2.CAR and seven co-expressed the C7R (Figure 1B). We visualized single, viable T cells in states of memory differentiation, activation, apoptosis regulation, proliferation, cytokine production, cytotoxicity, tissue infiltration, and exhaustion-related checkpoint expression (Figures 1C and 1D; Figure S4). We detected markers indicative of different subsets, including Th2, Th17, Th22 in CD4 T cells, and Tc2, Tc17, and Tc22 in CD8 T cells (Figures 1C and 1D; Figure S4). On average, GD2.CARTs express the CAR at 56.71% (range: 23.8%–89.7%), while C7R-GD2.CARTs co-express both the CAR and C7R at 23.07% (range: 10.4%–43.5%) (Table S3). T cell activation markers were upregulated upon stimulation with LAN-1 cells (Figures 1C and 1D) or with PMA and ionomycin (Figure S5). Overall, the evaluation of T cell characteristics demonstrates the functionality of the 33-marker FSFC panel, providing a foundation for subsequent assessments of differences among CART products.

### Assessment of variability in T cell activation across CART products

**Figure 2:**
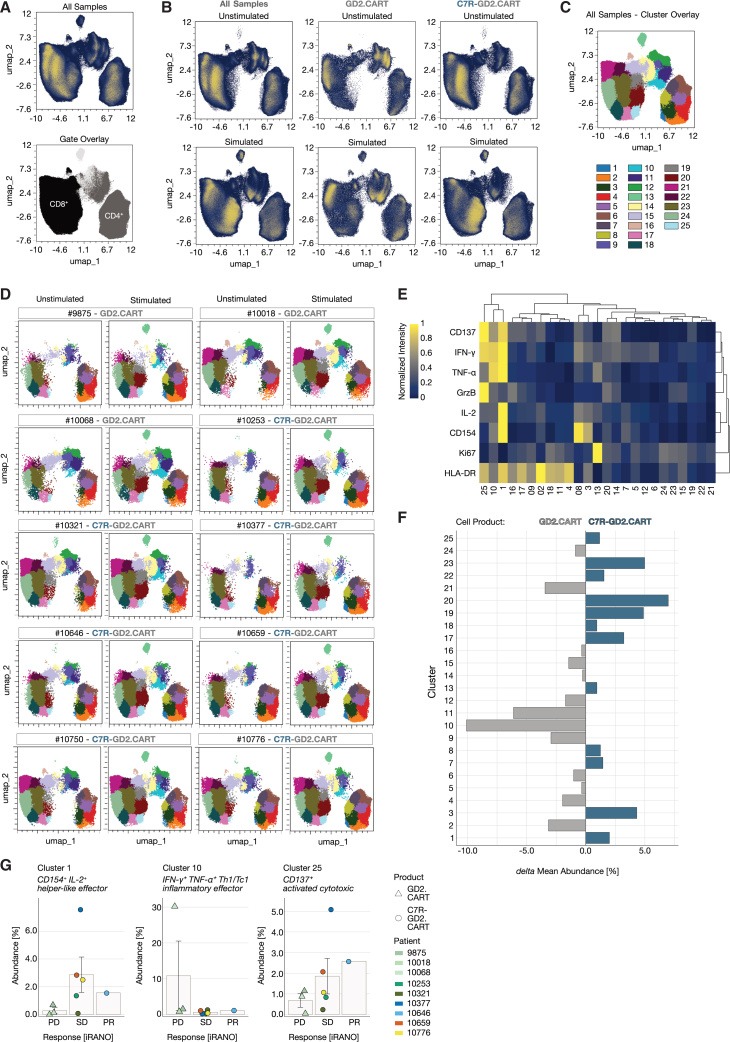
High dimensional analysis of CART product groups utilizing functional T cell markers (A) Uniform Manifold Approximation and Projection (UMAP) performed with max equal 100,000 CD3^+^ live T cells from each T cell product (*n* = 10) in LAN-1-stimulated and unstimulated condition (total *n* = 20) including all markers except CD3 and the viability dye used for pre-filtering and markers detecting the CAR and C7R (14g2a and CD34, respectively). Phenotypic distribution illustrated by overlaying manually set CD4 and CD8 gates. (B) Differences in stimulation and T cell products by displaying UMAP results of GD2.CART products (*n* = 3) and C7R-GD2.CART products (*n* = 7) separately. (C) Downstream of the UMAP, a FlowSOM clustering (k = 25) was performed on CD137, IFN-γ, TNF-α, GrzB, IL-2, CD154, HLA-DR, Ki67, umap-1, and umap-2. (D) Cluster distribution on UMAP between patients. (E) A composition heatmap with hierarchical clustering (average) shows protein abundance per cluster (min-max-scaling). (F) Cluster abundance comparison between cell products shows the delta mean abundance (%), calculated by subtracting the mean abundance of each cluster in the GD2.CAR product group (*n* = 7) from that of the GD2.CAR product group (*n* = 3). (G) Mean cluster abundance (%) for each patient is shown alongside the corresponding iRANO score for T cell products in patients with progressive disease (PD), stable disease (SD), or partial response (PR) after 6 weeks of treatment. Data are represented as mean ± SEM.

Using the panel described in Figure 1, we incorporated dimension reduction and clustering algorithms to visualize differences in T cell compositions among donors. Data cleaning was conducted with *flowCut*, and scaling was performed using arcsinh transformation. Single, live T cells were identified through manual gating (Figures 1C and 1D). To minimize donor bias, we standardized samples by subsampling to a maximum of 100,000 CD3^+^ live cells per donor. For dimension reduction, we applied Uniform Manifold Approximation and Projection (UMAP) analysis, organizing phenotypically similar events into distinct clusters. UMAP visualizations included LAN-1-stimulated and unstimulated samples, excluding features indicating viability, CD3, 14g2a (GD2.CAR), and CD34 (C7R). Differences in population distribution were observed between unstimulated and stimulated samples, as well as between GD2.CART products with and without C7R. Gates for CD4^+^ and CD8^+^ cells were applied to the UMAP to assist with population identification (Figures 2A and 2B).

To investigate potential associations between T cell activation states and clinical outcomes, we analyzed key activation markers (CD137, IFN-γ, TNF-α, GrzB, IL-2, CD154, HLA-DR, and Ki67) in relation to UMAP distributions (umap-1, umap-2). This approach identified 25 distinct clusters (Figure 2C), with variations in cluster abundance across individual patient samples (Figure 2D). The FlowSOM analysis revealed several activated T cell populations, specifically in clusters 1 (CD137, IFN-γ, TNF-α, IL-2, CD154, and HLA-DR), 10 (IFN-γ, TNF-α), and 25 (CD137, IFN-γ, GrzB, and HLA-DR) (Figure 2E). These clusters correspond to functionally distinct T cell subsets, which can be characterized as CD154^+^ IL-2^+^ helper-like effector T cells (cluster 1), IFN-γ^+^ TNF-α^+^ Th1/Tc1 inflammatory effector T cells (cluster 10), and CD137^+^ activated cytotoxic T cells (cluster 25). While clusters 1 and 25 were more prevalent in C7R-GD2.CARTs, cluster 10 was predominantly found in GD2.CART-only samples (Figure 2F). Cluster 10 was highly present in one cell product (10018) from patients with PD. Clusters 1 and 25 were most abundant in one cell product (10377) from patients with SD. None of the three clusters shows highest abundancy in the cell product of the patient with PR (Figure 2G). This analysis did not reveal clusters associated with tumor responses (PD, SD, and PR), suggesting that other factors may influence outcomes. However, this assessment did reveal variable abundance of distinct phenotypic clusters across donors, highlighting differences between stimulated and unstimulated conditions, as well as between GD2.CART and C7R-GD2.CART products.

### Unsupervised analysis reveals associations between T cell populations and clinical outcome

**Figure 3:**
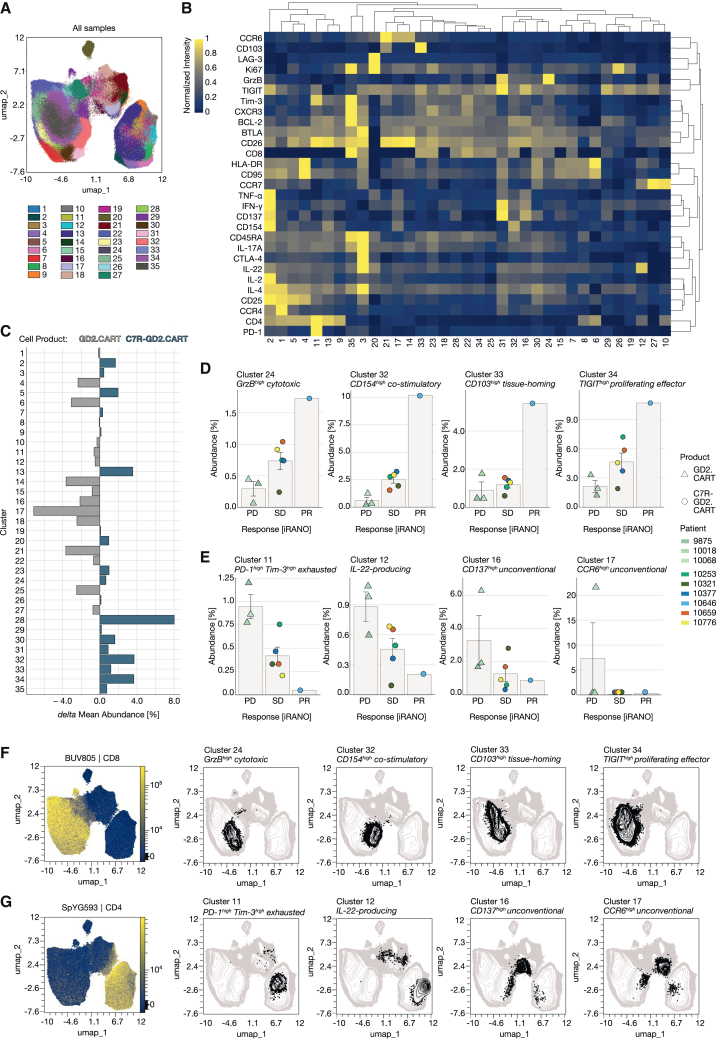
Unsupervised FlowSOM clustering of live T cells reveals marker compositions and cell product-specific differences in CART products (A) FlowSOM clustering performed with max equal 100,000 CD3^+^ alive cells from each T cell product (*n* = 10) in LAN-1-stimulated and unstimulated condition (total *n* = 20) including all markers except CD3 and the viability dye used for pre-filtering and markers detecting the CAR and C7R (14g2a and CD34, respectively). (B) A composition heatmap with hierarchical clustering (average) shows protein abundance per cluster (min-max-scaling). (C) Cluster abundance comparison between cell products shows the delta mean abundance (%), calculated by subtracting the mean abundance of each cluster in the C7R-GD2.CART cell product group (*n* = 7) from that of the GD2.CART cell product group (*n* = 3). Mean cluster abundance (%) for each patient is shown alongside the corresponding iRANO score for T cell products in patients with progressive disease (PD), stable disease (SD), or partial response (PR) after 6 weeks of treatment. Data are represented as mean ± SEM. The abundance of eight clusters is shown, representing (D) four clusters positively associated with clinical response and (E) four clusters negatively associated with clinical response. (F and G) Cells of the clusters are displayed in the UMAP (black), associating their location with CD4 and CD8 expressing cells represented by the fluorescence intensity of the respective antibodies (color continuous scale).

To minimize potential bias from activation-focused analyses, we applied an unsupervised FlowSOM clustering approach to live T cells from both unstimulated and LAN-1-stimulated conditions. This analysis identified 50 clusters, each defined by distinct marker expression patterns (Figure S6). To improve interpretability, these clusters were consolidated into 35 metaclusters based on marker similarities and spanning tree topology (Figures 3A and 3B). The resulting metaclusters are found in the main T cell populations (CD8^+^, CD4^+^, and CD4^−^CD8^−^, as supported by UMAP overlays) and correspond to their functional subsets, enabling biologically meaningful comparisons between GD2.CART-only and C7R-GD2.CART products. Differences in metacluster abundances between the product types are illustrated as delta abundances (Figure 3C). For a comparison between individual cell products, we examined associations between the identified clusters in LAN-1-activated T cells and corresponding clinical outcomes. Comparing the iRANO scores of each CART product with relative cluster frequencies revealed that four clusters (24, 32, 33, and 34) were positively associated with clinical outcomes (Figure 3D), showing the lowest abundance in cell products from patients with PD, increased abundance in those with SD, and the highest abundance in the in cell product of the partial responder after six weeks of treatment. In contrast, clusters 11, 12, 16, and 17 were more abundant in products from patients with poor outcome (Figures 3E and S7), with cluster 17 in particular accounting for 20% of the T cell composition in the product from one patient with PD. Notably, clusters positively associated with clinical responses (PD, SD, and PR) comprised predominantly CD8^+^ T cells (Figure 3F), whereas clusters negatively associated with clinical responses were found within the CD4^+^ or double-negative (CD4^−^CD8^−^) T cell populations (Figures 3G and S8), with elevated levels of exhaustion markers PD-1 and TIM-3 (cluster 11) or IL-22 expression (cluster 12) in CD4^+^ T cells and elevated levels of CD137 (cluster 17) and CCR6 (cluster 16) in unconventional CD4^−^CD8^−^ T cells. In summary, this analysis identified associations between T cell phenotypes and clinical outcomes, with clusters positively associated with clinical responses primarily found in CD8^+^ T cells.

### Image-based assessment of tumor killing activity in GD2.CART products

**Figure 4:**
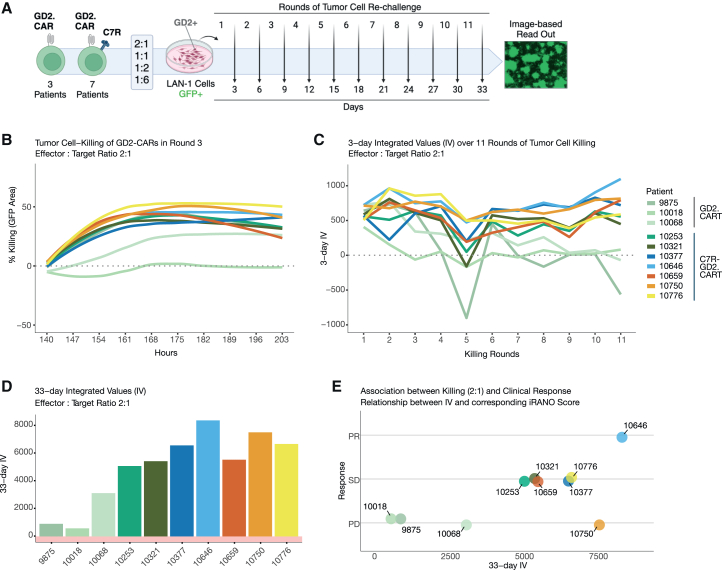
Imaging-based assessment of serial killing assays over 33 days and its association with clinical response of CART products (A) Schematic workflow illustrating the serial killing assay performed using live cell imaging over 33 days. T cell products were challenged every 3 days at effector-target ratios of 2:1, 1:1, 1:2, and 1:6 with a GD2-positive LAN-1 tumor cell line expressing GFP for a total of eleven rounds. Images were taken every 7 h and evaluated based on the total area of GFP-positive cells. Created with BioRender.com (B) Line graph depicting tumor cell-killing as indicated by the loss of GFP^+^ area (% killing). CART products are color-coded, effector-target ratio of 2:1 over time and serial killing round 3 are shown. (C) The serial killing dynamics of each T cell product are compared using a 3-day integrated value (IV) at effector-target ratio 2:1, determined by summing the killing percentages of each CAR-T cell product for each round, represented as a line graph over a 33-day period. (D) A 33-day IV, determined by summing the 3-day-IV of each CAR-T cell product at effector-target ratio 2:1 for all rounds, represented as bar graphs. (E) Association between killing and clinical response is shown by comparing the 33-day IV of the T cell products at effector-target ratio 2:1 with the iRANO score of the respective patient.

To evaluate the long-term tumor-killing capacity of GD2.CART products in a 33-day serial killing assay, CART products were co-cultured with GFP-expressing LAN-1 tumor cells at effector-to-target ratios of 2:1, 1:1, 1:2, and 1:6, with repeated tumor cell exposures every three days (Figure 4A). Tumor cell killing was quantified by measuring the remaining GFP^+^ area over time (Figure 4B). To assess killing efficiency over repeated exposure cycles, we calculated a 3-day integrated value (IV) by summing tumor cell elimination over 7-h intervals for each round and effector-to target ratio. Serial killing dynamics were visualized as line graphs (Figure 4C), while the cumulative 33-day IV, calculated by summing all 3-day IVs over 33 days, provided a serial killing score for each CART product at each effector-to-target ratio (Figures 4D and S9A). This analysis identified products 9875, 10018, and 10068, lacking C7R (Table S3), as having the lowest tumor-killing capacity with a 33-day iV of 906, 580, 3122, respectively. A positive association was observed between the serial killing capacity of cell products and clinical outcomes (iRANO scores), with the cell product from the patient with PR showing a higher 33-day IV of 8379 compared to those from patients with SD at 5084, 5428, 6572, and 5540, which in turn had higher IV scores than those from patients with PD. Product 10750 represents an exception, as it demonstrated strong *in vitro* killing (7517) despite the patient displaying PD in the clinical trial (Figures 4E and S9B). Of note, this patient’s tumor was later found to be GD2 negative before treatment despite H3K27 alteration which likely explained the lack of clinical response, regardless of CART potency. Overall, this image-based assay revealed substantial variability in tumor-killing efficiency among GD2.CART products, with notable differences associating with clinical outcomes.

### Integration of FSFC and serial killing results highlights functional heterogeneity in CART products

**Figure 5:**
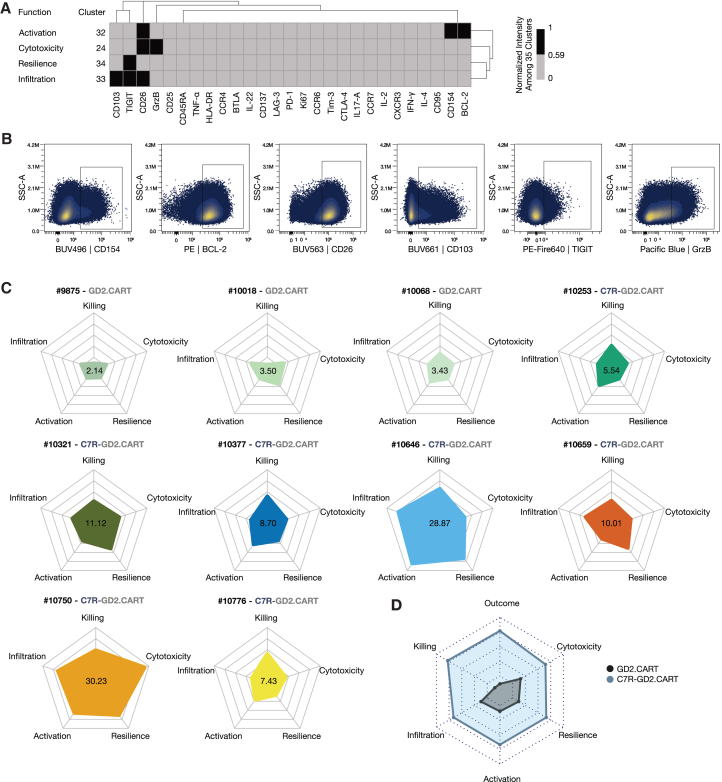
Integrated FSFC and serial killing assays reveal functional heterogeneity and potency of CART products (A) Protein abundance in the CD8^+^ T cell clusters 24, 32, 33, and 34 positively associated with response is represented using the initial cluster-scaling of the marker composition analysis (Figure 3B), with a threshold value of 0.58 indicating protein presence (black). The clusters exhibited distinct profiles with higher expression of GrzB, CD26 (cluster 24); BCL-2, CD26, and CD154 (cluster 32); CD103, TIGIT, CD26 (cluster 33); and TIGIT (cluster 34), suggesting roles in T cell cytotoxicity, activation, tissue infiltration, and resilience, respectively. (B) Gating strategy shown for boolean gates applied to single, viable CD3^+^ CD8^+^ cells for each cluster-based marker expression displayed in panel A. (C) Spider charts visualize multivariate data for each T cell product, allowing performance assessment across the displayed T cell features. The values within each chart represent the calculated area of the polygon formed by the z-scaled metrics, expressed in arbitrary units. Higher area values indicate a greater overall performance. (D) A summary chart (*n* = 10) compares the functional performance of the GD2.CART and C7R-GD2.CART products by displaying the mean of each T cell feature per cell product group.

To assess the functional heterogeneity of GD2.CART products, we integrated phenotypic profiling from FSFC with tumor-killing data from the serial killing assays. To facilitate comparison between clusters identified in the FSFC data of the CART products, the mean fluorescence intensity of each marker was normalized across all 35 clusters (Figure 3B). Among these, four CD8^+^ clusters (24, 32, 33, and 34) were enriched in T cell products linked to SD and showed the highest abundance in the T cell product linked to the best clinical response (PR) (Figures 3D and 3F). Those four CD8^+^ T cell clusters were characterized by prominent expression of proteins linked to effector attributes: cytotoxicity, activation, infiltration, resilience. Cluster 24 was characterized by higher expression of GrzB and CD26, suggesting a role in T cell cytotoxicity. Cells in cluster 32 expressed higher BCL-2, CD26, and CD154, indicating potential for T cell activation. Cluster 33 exhibited elevated CD26, TIGIT, and CD103, suggesting a role in T cell infiltration into tissues, while cluster 34 showed relatively higher TIGIT levels, indicative of enhanced resilience (Figure 5A; Table S1). When comparing treatment duration among CART-treated patients, the same four clusters were present in intermediate and long responders (Figure S10; Table S4). Furthermore, when analyzing the abundances of the response-associated clusters alongside cytokine changes measured in the peripheral blood of patients post-treatment, the response-negative-associated clusters exhibit an opposite correlation pattern compared to the response-positive-associated clusters (Figures S11A–S11F). This distinct correlation suggests that the clusters represent different functional roles in the dynamics of the post-treatment immune response. To assess the frequency of cells with functional attributes associated with the four clusters linked to clinical response in the individual CART products, we gated on single, viable CD3^+^CD8^+^ T cells expressing the relevant marker combinations from each cluster (Figure 5B). These frequencies were integrated with the results from the killing assay and visualized in spider charts, with larger shapes representing higher functional capacity (Figure 5C). Four axes display T cell frequencies (Figure 5B), while one axis represents the 33-day IV from the serial killing assay at an effector-to-target ratio of 2:1 (Figure 4D). Based on our analysis, these five T cell attributes are associated with improved clinical outcomes of CART products in this cohort. Notably, the GD2.CART products 9875, 10018, and 10068, lacking C7R, displayed the lowest functionality across multiple attributes. Conversely, the C7R-GD2.CART cell products 10646 and 10750 (tumor GD2^-^) demonstrated high functionality, marked by superior tumor-killing capacity and potential for tissue infiltration, resilience, and activation. When comparing GD2.CART products with those with C7R-GD2.CART and incorporating therapeutic outcomes from iRANO scores (outcome), C7R-GD2.CART products displayed a more robust functional phenotype than those expressing the GD2.CAR alone (Figure 5D). We further explored whether the functional signatures of the autologous products correlate with *in vivo* CAR transgene persistence measured by qPCR over 4 weeks post-infusion. Analysis revealed no significant association (r = −0.07), suggesting that phenotypic characteristics associated with clinical response do not associate with high persistence capacity observed in patients with SD in this cohort (Figures S10 and S11G). In summary, integrating FSFC analysis with the killing assays enabled a pertinent characterization of CART products facilitating a functional comparison.

## Discussion

We established a 33-marker FSFC panel and integrated it with functional assays to characterize CART products. Our approach enabled an extensive analysis of T cell phenotypes and functional diversity in GD2.CARTs and C7R-GD2.CARTs from a phase 1 clinical study targeting GD2-positive brain tumors. Compared to GD2.CART only products, we identified increased numbers of T cells with cytotoxicity, activation, resilience, and tissue infiltration characteristics in C7R-GD2.CART products, with the highest abundance in the patient showing partial responses. Serial tumor-killing assays supported the superior cytotoxic potential of C7R-GD2.CARTs, associating with improved therapeutic efficacy. To summarize the functional distinctions among CART products, we employed spider charts that effectively highlight the differences in key T cell attributes. The visualized differences, with larger shapes representing higher functional capacities, confirmed the superior function of C7R-GD2.CARTs compared to GD2.CARTs, associating with improved clinical outcomes. While small patient numbers and the single objective response limit this interpretation, the associations identified warrant validation in larger studies. In addition, exploratory correlation analyses linked infusion product composition to systemic cytokine dynamics but did not reveal strong correlations with *in vivo* CART cell persistence. This indicates that while product features can shape early immune responses, clinical benefit is not solely dependent on persistence and may involve additional mechanisms. This small model cohort serves as a foundation for applying our FSFC panel and highlighting its potential applicability in clinical interpretation. Larger studies are essential to introduce statistical power and to investigate generalizability. By expanding the patient population, we can validate the effectiveness of the FSFC panel and gain a clearer understanding of its utility across diverse clinical settings.

In attempts to identify CART characteristics that correlate with clinical efficacy, methods such as scRNA-seq and conventional flow cytometry have captured the heterogeneity of T cell products and identified clinically relevant features.^11^^,^^12^ However, these methods often fall short due to insufficient cell numbers, limitations in marker inclusion, complex bioinformatics or time, and cost constraints,^13^ reducing their usefulness for routine assessment in clinical trials. FSFC bridges this gap by enabling a high-dimension analysis while remaining practical for routine use in clinical settings. Our analysis revealed heterogeneity in CART characteristics across infusion products, associating with variations in clinical responses. While targeted analysis approaches focusing on predefined markers provide valuable insights, they may lead to incomplete assessments of T cell functionality.^14^ Our unsupervised analytical approach enhanced the characterization of T cell products by uncovering functional relationships but may not capture all factors influencing efficacy. Importantly, the FSFC panel allows marker interchangeability to meet specific research needs or clinical applications and is applicable to any T cell therapy product.

By employing classical gating strategies alongside unsupervised high-dimensional analyses, we identified four T cell features linked to clinical benefit: activation, infiltration capacity, cytotoxicity, and resilience. Activation is exemplified by a CD8^+^ T cell subpopulation expressing CD154 and CD26, which is associated with improved clinical responses. While CD154 is known to enhance T cell activation and anti-tumor responses,^15^ the co-expression of CD154 and CD26 in CARTs or tumor-specific CD8^+^ T cells has not been extensively studied. CD26, a costimulatory receptor, enhances T cell cytotoxicity and interacts with the extracellular matrix, hence may contribute to infiltration and anti-tumor immunity.^16^^,^^17^ We observed a CD8^+^CD103^+^ cluster prominent in the cell product of the patient with the best therapy response (10646), indicating a potential link between infiltration capacity and treatment outcomes that merits further exploration. With respect to cytotoxicity, we identified a GrzB^+^ CD8^+^ T cell subpopulation linked to improved patient responses, reinforcing its utility as a performance indicator for evaluating T cell product efficacy.^18^^,^^19^ The immunosuppressive TME can impair T cell function via immune checkpoint activation, including TIGIT expression.^20^ Interestingly, our analysis revealed a subset of CD8^+^ T cells expressing higher levels of TIGIT that was positively associated with clinical outcomes, suggesting a more complex role for this marker in mediating T cell resilience within the clinical products.^21^

We designed the evaluation framework to facilitate CART product characterization for clinical trials. The quality and function of CARTs are influenced by manufacturing processes, pre-treatment regimens, and patient health, all of which shape the T cell repertoire and impact therapeutic efficacy.^22^ Our FSFC panel offers a framework to assess these factors, providing insights into CART performance across various clinical settings. Here, T cell functionality was validated by an image-based serial killing assay that mirrored the clinical course, except for one patient (10750) whose GD2-targeting T cell product exhibited strong *in vitro* cytotoxicity but failed clinically, likely due to the presence of the tumor retrospectively identified as negative despite the presence of the H3K27 mutation.^3^ While functional assays such as the 30-day serial killing assay provide valuable insights into cytotoxic potential, FSFC allows for rapid profiling of T cell products, enabling timely decision-making in clinical applications. By improving our understanding of T cell functionality and adaptability with our 33-marker FSFC panel, we thus contribute to ongoing efforts to optimize CART therapy and advance precision medicine in oncology.

### Limitations of the study

This study serves as a foundational model, and further validation in larger patient populations is essential to enable statistically powered analyses and confirm the broader applicability of the FSFC panel across diverse clinical contexts. The small cohort size, with only one partial responder, restricts generalizability and may not capture the full complexity of therapeutic efficacy. While our unsupervised analytical approach helps reduce bias, it may not account for all variables influencing treatment success. Exploratory correlation analyses did not reveal strong links between infusion product composition and *in vivo* CAR T cell persistence, suggesting that other mechanisms contribute to clinical benefit beyond those assessed by the current FSFC panel. Lastly, one case highlighted a discrepancy between *in vitro* cytotoxicity and clinical outcome, where a product demonstrated strong laboratory performance but failed clinically due to absence of the target antigen. This underscores the limitations of relying solely on laboratory assays to predict therapeutic efficacy in clinical settings.

## Resource availability

### Lead contact

Further information and requests for resources and reagents should be directed to and will be fulfilled by the lead contact, Michael Schmueck-Henneresse (michael.schmueck-henneresse@bih-charite.de).

### Materials availability

This study did not generate new unique reagents. Patient-derived (C7R) GD2.CAR T cell products were obtained as part of the GAIL-B clinical trial (ClinicalTrials.gov/study/NCT04099797 identifier: NCT04099797) and are not available for distribution.

### Data and code availability

This paper does not report original code or new protein structural data. All data needed to evaluate the conclusions are provided in the paper and/or the supplemental information. Details of flow cytometry panel design and optimization are included in the supplemental information. All datasets generated and analyzed in this study, including raw FSFC files and source data for the figures, are available from the corresponding author upon reasonable request.

## Acknowledgments

We thank the patients that agreed to participate in this study for their donations.

The study was supported in parts by 10.13039/501100002347German Federal Ministry of Education and Research (CONAN, 16GW0328K) provided by the 10.13039/501100002347Federal Ministry of Education and Research (BMBF). 10.13039/501100002347German Federal Ministry of Education and Research (BIH Center for Regenerative Therapies, 13353 Berlin – M.S.-H.). Research grants by the Einstein Center for Regenerative Therapies (S.S. and M.S.-H.). 10.13039/100004917Cancer Prevention and Research Institute of Texas (RP-240473), The Faris Foundation, Chance for Hope Foundation, ChadTough Defeat DIPG Foundation, and VioletFoundation for Pediatric Brain Cancer (B.O.). The funders had no role in study design, data collection and analysis, decision to publish, or preparation of the manuscript.

## Author contributions

Writing - original draft: S.S. and M.S.-H.; conceptualization: S.S. and M.S.-H.; investigation: S.S., M.F.-S., M.L., L.E., S. Schlickeiser, S.P., L.P., C.L.T., C.M.R., B.O., F.L., J.K., and D.K.; writing - review and editing: S.S., C.M.R., B.O., and M.S.-H. methodology: S.S., M.F.-S., M.L., M.M., J.K., and D.K.; resources: B.O. and M.S.-H.; funding acquisition: B.O. and M.S.-H.; data curation: S.S., M.L., and L.E. validation: S.S. and M.S.H. software: no software was developed in the context of this study. supervision: M.S.-H.; formal analysis: S.S., M.F.S., M.L., L.E., and S Schlickeiser; project administration: M.S.-H; visualization: S.S., M.L., and L.E.

## Declaration of interests

The authors declare no competing interests.

## Declaration of generative AI and AI-assisted technologies in the writing process

During the preparation of this work the author(s) used Claude by Anthropic (claude.ai) in order to restructure and refine sentences and paragraphs for clarity and flow. After using this tool/service, the authors reviewed and edited the content as needed and take full responsibility for the content of the publication.

## STAR★Methods

### Key resources table

**Table:** 

REAGENT or RESOURCE	SOURCE	IDENTIFIER
**Antibodies**		

Spark UV387 anti-human CD45RA, clone HI100	Biolegend	Cat#304179; RRID: AB_2922538
Mouse Anti-Human CD25.Star Bright UltraViolet 445, clone MEM-181	BioRad	Cat#MCA2127SBUV445; RRID: AB_3100638
BUV563 mouse anti-human CD26, clone M-A261	BD Biosciences	Cat#749318; RRID: AB_2873692
BUV661 Mouse anti-human CD103, clone Ber-ACT8	BD Biosciences	Cat#749993; RRID: AB_2874215
Spark YG 593 anti-human CD4, clone SK3	Biolegend	Cat#344672; RRID: AB_2894479
BUV805 mouse anti-human CD8, clone RPA-T8	BD Biosciences	Cat#749366; RRID: AB_2873737
Brilliant Violet 421 anti-human CD197 (CCR7), clone G043H7	Biolegend	Cat# 353208; RRID: AB_11203894
Anti-human 14g2a, clone 1A7	Provided by Bilal A. Omer (Center for Cell and Gene Therapy, Texas Children’s Hospital, Houston Methodist Hospital, Baylor College of Medicine, Houston, TX, USA; Texas Children’s Cancer and Hematology Centers, Texas Children’s Hospital, Baylor College of Medicine, Houston, TX, USA)	–
BV480 Mouse anti-human CD34, clone 563	BD Biosciences	Cat#746415; RRID: AB_2743726
Brilliant Violet 605 anti-human CD152 (CTLA-4), clone BNI3	Biolegend	Cat#369610; RRID: AB_2632779
Brilliant Violet 650 anti-human CD366 (Tim-3), clone F38-2E2	Biolegend	Cat#345028; RRID: AB_2565829
Brilliant Violet 711 anti-human CD196 (CCR6), clone G034E3	Biolegend	Cat#353436; RRID: AB_2629608
Brilliant Violet 785 anti-human CD279 (PD-1), clone EH12.2H7	Biolegend	Cat#329930; RRID: AB_2563443
Spark Blue 550 anti-human CD3, clone SK7	Biolegend	Cat#344852; RRID: AB_2819985
PE/Dazzle 594 anti-human CD272 (BTLA), clone MIH26	Biolegend	Cat#344522; RRID: AB_2687142
PE/Fire640 anti-human TIGIT (VSTM3), clone A15153G	Biolegend	Cat#372744; RRID: AB_2922588
PE/Fire700 anti-human CD194 (CCR4), clone L291H4	Biolegend	Cat#359436; RRID: AB_2894491
PE/Fire810 anti-human HLA-DR, clone L243	Biolegend	Cat#307683; RRID: AB_2904336
Alexa Fluor 647 anti-human CD95 (Fas), clone DX2	Biolegend	Cat#305618; RRID: AB_528893
APC/Fire810 anti-human CD183 (CXCR3), clone G025H7	Biolegend	Cat#353762; RRID: AB_2904376
Alexa Fluor 488 anti-human IL-4, clone MP4-25D2	Biolegend	Cat#500817; RRID: AB_493324
APC/Fire750 anti-human IFN-g, clone 4S.B3	Biolegend	Cat#502548; RRID: AB_2572107
BUV496 Mouse Anti-Human CD40L (CD154), clone 24-31	BD Biosciences	Cat# 752853; RRID: AB_2917808
BUV737 Rat anti-human IL-2, clone MQ1-17H12	BD Biosciences	Cat#612836; RRID: AB_2870158
Brilliant Violet 570 anti-human IL-17A, clone BL168	Biolegend	Cat#512324; RRID: AB_2563886
Brilliant Violet 750 anti-human Ki-67, clone ki-67	Biolegend	Cat#350536; RRID: AB_2910400
NovaFluor Blue 660-120S anti-human CD223 (LAG-3), clone 3DS223H	Thermo Fisher Scientific	Cat#H048T03B08; RRID: AB_2925963
PacificBlue anti-human/mouse Granzyme B Recombinant, clone QA16A02	Biolegend	Cat# 372218; RRID: AB_2728385
PE anti-Bcl-2, clone 100	Biolegend	Cat#658708; RRID: AB_2563282
PE/Cyanine5 anti-human CD137 (4-1BB), clone 4B4-1	Biolegend	Cat#309808; RRID: AB_830670
PE-Cy7 Mouse Anti-Human IL-22, clone MH22B2	BD Biosciences	Cat#567579; RRID: AB_2916652
PerCP anti-human TNF-a, clone MAb11	Biolegend	Cat#502924; RRID: AB_2561288

**Biological samples**		

Patient-derived autologous (C7R)GD2.CAR T cell products	Lin et al.^3^	ClinicalTrials.gov identifier: NCT04099797

**Chemicals, peptides, and recombinant proteins**		

BD HorizonTM Brilliant Stain Buffer	BD Biosciences	Cat#563794
Human TruStain FcX	Biolegend	Cat#422302
Zombie NIR Fixable Viability Kit	Biolegend	Cat#423106
eBioscienceTM Foxp3/Transcription Factor Staining Buffer Set	Invitrogen	Cat#00-5523-00
Ultracomp eBeads Kompensations-Beads	Invitrogen	Cat#01-2222-42

**Critical commercial assays**		

Alexa Fluor 700 Conjugation Kit (Fast) - Lightning-Link	Abcam	Cat#ab269824

**Deposited data**		

Cytokine data (Luminex)	Lin et al.,^3^ Provided by Bilal A. Omer (Center for Cell and Gene Therapy, Texas Children’s Hospital, Houston Methodist Hospital, Baylor College of Medicine, Houston, TX, USA; Texas Children’s Cancer and Hematology Centers, Texas Children’s Hospital, Baylor College of Medicine, Houston, TX, USA)	N/A
Cell product persistence data (cell frequencies, flow)	Lin et al.,^3^ Provided by Bilal A. Omer (Center for Cell and Gene Therapy, Texas Children’s Hospital, Houston Methodist Hospital, Baylor College of Medicine, Houston, TX, USA; Texas Children’s Cancer and Hematology Centers, Texas Children’s Hospital, Baylor College of Medicine, Houston, TX, USA)	N/A

**Experimental models: Cell lines**		

LAN-1 cells (WT)	Provided by Bilal A. Omer (Center for Cell and Gene Therapy, Texas Children’s Hospital, Houston Methodist Hospital, Baylor College of Medicine, Houston, TX, USA; Texas Children’s Cancer and Hematology Centers, Texas Children’s Hospital, Baylor College of Medicine, Houston, TX, USA)	N/A
LAN-1-GFP cells	Provided by Bilal A. Omer (Center for Cell and Gene Therapy, Texas Children’s Hospital, Houston Methodist Hospital, Baylor College of Medicine, Houston, TX, USA; Texas Children’s Cancer and Hematology Centers, Texas Children’s Hospital, Baylor College of Medicine, Houston, TX, USA)	N/A

**Software and algorithms**		

CellReporterXpress software	Molecular Devices	RRID:SCR_025681
SpectroFlo software, v3.2.1	Cytek	RRID:SCR_025494
FlowJo, v10.9.0	BD Biosciences	RRID:SCR_008520
OMIQ, accessed in 2024	Dotmatics	app.omiq.ai
GraphPad Prism, version 9	GraphPad Software	RRID:SCR_002798
OpenAI, 2023, accessed in 2024	Ecosia Chat	https://www.ecosia.org/chat
R Statistical Software, v4.4.0	R Core Team	RRID:SCR_001905

### Experimental model and subject details

#### Patient samples

The clinical trial was approved by the Baylor College of Medicine biosafety committee and institutional review board, and the US Food and Drug Administration (ClinicalTrials.gov identifier: NCT04099797). Written informed consent (and assent when applicable) was obtained for all patients including sharing samples for research analysis. The study was conducted in accordance with the Declaration of Helsinki. For the establishment of the FSFC panel, freshly isolated peripheral blood mononuclear cells (PBMCs) from heparinized whole blood samples of healthy donors were cultured. The study was approved by the Ethics Committee of the Charité (EA4/091/19). Ten CART products were analyzed, all originating from the phase 1 clinical trial GAIL-B targeting patients with GD2-positive brain tumors. All samples were de-identified before shipment and analysis. The GAIL-B study evaluated the application and tolerability of the co-receptor C7R in GD2.CART cells, based on promising results from prior *in vitro* and murine studies that supported CART functionality. Within the framework of GAIL-B, three patients (9875, 10018, 10068) were treated with GD2.CARTs, while seven patients (10253, 10321, 10377, 10646, 10659, 10750, 10776) received C7R-GD2.CARTs. Autologous CART products were administered intravenously, and clinical efficacy was determined using the iRANO score, assessing tumor size after six weeks. Cytokine levels and CART persistence data were acquired from patients' peripheral blood samples collected pre-treatment, 3 h, 1 week, 2 weeks, and 4 weeks post-treatment as previously described.^3^

### Method details

#### Cell preparation

The analyzed samples were derived from coded cryopreserved autologous CART products in the GAIL-B trial that were excess to clinical use. The cell products were transported on dry ice and stored in the gas phase of liquid nitrogen until analysis. On the day of the experiment, the CART products were thawed and cultured at 37°C in a 5% CO_2_ atmosphere using CTL medium, which consisted of 50% Advanced RPMI 1640 (PAN-Biotech), 50% Clicks medium (Fujifilm, Irvine Scientific), and 2 mM L-GlutaMAX (Gibco, Thermo Fisher Scientific) supplemented with 10% fetal calf serum (FCS, PAA). For the establishment of the FSFC panel, freshly isolated PBMCs from heparinized whole blood samples from healthy donors were cultured in RPMI 1640 medium (Thermo Fisher Scientific) supplemented with 10% FCS and 1% penicillin-streptomycin (Biochrom). The blood collection was approved by the Charité – Universitätsmedizin Berlin Ethics Committee (EA4/091/19).

#### T cell activation

For a CAR-specific activation, C7R-GD2.CART and GD2.CART products were incubated for 12 h with the GD2^+^ LAN-1 neuroblastoma tumor cell line (Sigma-Aldrich). LAN-1 cells were cultured in Dulbecco’s Modified Eagle Medium (DMEM) (4.5 g/L glucose, Thermo Fisher Scientific) with 10% FCS and 2 mM L-GlutaMAX in T75 cell culture flasks and split every three to four days in a ratio of 1:2 after reaching a confluence of 80%. For stimulation, 0.5∗10^6^ LAN-1 cells were co-cultured in a 24-well plate in 0.5 mL of CTL medium at an effector to target (E:T) ratio of 1:1. PBMCs were activated via plate-bound 1 μg/mL anti-CD3 (Invitrogen) and 1 μg/mL anti-CD28 (Biolegend) monoclonal antibodies. Cells were cultured with 10 ng/mL IL-7 and IL-15 (human, CellGenix) (expanded T cells). To measure activation markers and cytokines, CART products, PBMCs and expanded T cells were activated in the presence of 2 ng/mL PMA and 0.5 μg/mL ionomycin (both Sigma-Aldrich) at 37°C and 5% CO_2_ in air. After 1 h, 2 μg/mL of the protein transport inhibitor Brefeldin A (Sigma-Aldrich) was added. The activation was stopped after 16 h by adding PBS. The cells were cooled to 4°C and stained for flow cytometry measurement.

#### Flow cytometry

Staining of GD2.CART products and PBMCs was conducted in 5 mL tubes using the eBioscience FoxP3/Transcription Factor Staining Buffer Set (Thermo Fisher Scientific). Prior to staining, cells were washed twice with PBS and stained with a viability dye (Zombie NIR, Biolegend, 1:3000 diluted) for 20 min. The samples were washed with PBS and an Fc block (Human TruStain FcX, Biolegend) was performed for 5 min. Staining was performed in brilliant stain buffer (BD Bioscience) for at least 20 min at 4°C. Extracellular staining was performed using the dyes stated in Table 1. After washing twice with PBS, the cells were fixed and permeabilized for 60 min at 4°C. After washing twice with permeabilization buffer (PB), intracellular staining was performed using the dyes stated in Table 2. The samples were washed twice with PBS, resuspended in PBS supplemented with 0.5% Bovine Serum Albumin and 2 mM EDTA, and acquired at a 5-laser full spectrum flow cytometer (Cytek Aurora).

The 33-color FSFC panel was established using PBMCs and expanded T cells from healthy individuals, following established protocols.^10^ Antibodies were titrated on activated or rested PBMCs. The optimal antibody dilution was determined based on distinct positive and negative populations and the highest stain index (SI), calculated as SI = (MFI_positive - MFI_negative)/(2 ∗ SD_negative), where MFI represents the mean fluorescence intensity and SD is the standard deviation of the respective population. Spectral unmixing was performed using compensation beads (UltraComp eBeads, Invitrogen) or single-stained cells, while references for antibodies detecting the GD2.CAR (14g2a) and the C7R (CD34) were established using C7R-GD2.CARTs. The compatibility of fluorochrome combinations was evaluated using the Complexity Index, a metric developed by Cytek. This index reflects the diversity of fluorescent markers and their intensities, calculated based on the ratio of spectral similarities between signatures, with a lower complexity index signifying a panel that is practical. Fluorescence minus one (FMO) staining assessed panel consistency in activated T cells from healthy donors (*n* = 3), showing little variation in marker-positive cell frequencies, with standard deviations below 8% for all markers tested (Figure S3).

#### Serial killing assay

A serial killing assay was performed to assess the killing capacity of T cell products against GD2-expressing LAN-1 cells. Thawed T cell products were incubated at distinct ratios (2:1, 1:1, 1:2, 1:6) with 4∗10^5^ GFP-positive LAN-1 cells at 37°C and 5% CO_2_. Every three days, a new round of killing was initiated by adding fresh LAN-1 cells in the same ratio, resulting in a total assay duration of eleven rounds over 33 days. Total area of GFP fluorescence for each condition was measured every 7 h using the ImageXpress Pico device (Molecular Devices) and total GFP area was quantified with CellReporterXpress software (Molecular Devices) with a 96-well clear bottom imaging plate (PhenoPlate # 6055302, Revvity).

### Quantification and statistical analysis

#### Flow cytometry data

FSFC data were unmixed and corrected in the SpectroFlo software (v3.2.1; Cytek) and in FlowJo (v10.9.0; BD Biosciences). Unmixed FCS files were analyzed in OMIQ (Dotmatics, accessed in 2024). Scaling was performed using arcsinh transformation. Data cleaning was performed via FlowCut on all features under the following settings: Segment = 500, Max Contin = 0.1, Mean of Means = 0.13, Max of Means = 0.15, Max of Valley Height = 0.1, Max Percent to Cut = 0.3, Low Density Removal = 0.1, no Gate Line was set, Max Channel for Mean Range = 1, Max Channel for Mean SD = 2, no Flagged Rerun was allowed, Uniform of Time Check = 0.22, and Removal Multi SD = 7.

For high-dimensional data analysis, subsampling was performed to include a maximum of 100,000 viable CD3^+^ lymphocytes per sample to reduce bias. For dimension reduction, UMAP was employed on LAN-1-stimulated and unstimulated samples from all patients, analyzing all features except time, scatter, viability dye, CD3, 14g2a, and CD34. The following parameters were used: number of neighbors = 15, minimum distance = 0.4, components = 2, metric = “euclidean,” learning rate = 1, epochs = 200, random seed = 2553, and embedding initialization = “spectral.” FlowSOM analysis was conducted on LAN-1-stimulated and unstimulated samples from all patients with k-values set to 25 or 50 as indicated. The parameters used were: xdim = 10, ydim = 10, rlen = 10, metric = “euclidean,” and random seed = 3421. A spanning tree analysis of the FlowSOM clusters, alongside UMAP locations, was conducted to visualize relationships and refine clusters. Heatmaps comparing marker expression levels across clusters were generated to support cluster characterization.

Manual gates were set to identify populations positive for the markers indicated. Population frequencies and mean fluorescence intensities of the fluorochromes investigated were exported for visualization.

#### Serial killing assay data

The data from the killing assay was analyzed in R Statistical Software (v4.4.0; R Core Team). Coding in R was assisted by Ecosia Chat, an AI language model developed by OpenAI (Ecosia Chat, 2023). Initially, the data were cleaned for consistency using the janitor package^23^ (v2.2.0). Merging with well-meta data were done using the readxl package^24^ (v1.4.3) and the dplyr package^25^ (v1.1.4). Normalized total areas were calculated by dividing the values at each subsequent timepoint by those at the initial timepoint (t1), allowing a comparative analysis of tumor cell-viability across different timepoints. The percentage of killing was determined based on the normalized total area values (100 - ((area/area^t1^)∗100)). Timepoints were converted into hours, with a duration of 7 h for each timepoint.

For calculating the integrated value, the data were filtered by the E:T (effector to target) ratios and grouped by donor. The 3-day integrated value (IV) was computed by summing the percentage killing values for each T cell product. The 33-day IV was computed by summing the 3-day IVs for each T cell product.

#### Cytokine and CART persistence analysis

For cytokine correlation analysis, Spearman correlation was performed to assess the relationship between the abundance of response-associated immune cell clusters and functional cytokine scores. Cytokines were functionally grouped into biologically relevant categories based on their known biological functions, and correlations were calculated for delta values between pre-treatment to week 1, pre-treatment to week 4, and week 1 to week 4 time points. For persistence analysis, CAR transgene levels were quantified by qPCR and area under the curve (AUC) over the measurement timepoints was calculated using trapezoidal integration. The relationship between radar chart polygon area and CAR transgene persistence AUC was analyzed using Pearson correlation analysis with linear regression modeling and 95% confidence intervals. Statistical significance was set at *p* < 0.05.

#### Data visualization

##### Flow cytometry data

Flow cytometry graphs were generated in OMIQ or FlowJo. Graphical abstract and schematic illustrations were created using BioRender.com. Standard deviations marker frequencies within fluorescence minus one (FMO) samples were displayed with GraphPad Prism (version 9, GraphPad Software, Boston, Massachusetts USA). Visualization of fluorochrome intensities and cluster abundancies was performed in R (v4.4.0; R Core Team). The dplyr package^25^ (v1.1.4) was used to prepare the data for visualization. Heatmaps were generated using the pheatmap package^26^ (v1.0.12). A min/max scaling to the data were performed before creating the heatmap to visualize fluorochrome intensity across the clusters. Hierarchical clustering was performed using the base R hclust function on the scaled data. The viridis package^27^ (v0.6.5) was employed for color mapping, while the readr package^28^ (v2.1.5) facilitated data import. For statistical output, the broom package^29^ (v1.0.9) was utilized, and the writexl package^30^ (v1.5.0) was used to export results.

Graphs were generated using the ggplot2 package^31^ (v3.5.1). The delta mean abundance of clusters was illustrated by showing the result of the abundance of each cluster in T cell products with the C7R subtracted by the abundance of each cluster in T cell products without the C7R (GD2.CAR only). To adjust axis labels, the scales package^32^ (v1.3.0) was employed.

Radar charts were created to visualize data from the ten T cell products using the fmsb package^33^ (v0.7.6). The data were z-scaled with the dplyr package^25^ (v1.1.4) to standardize axes for metrics. Custom color palettes were applied using the colormap package^34^ (v0.1.4) for visual distinction.

##### Serial killing assay

Plots were created using the ggplot2 package^31^ (v3.5.1). Line graphs visualize the relationship between time (in hours) and percentage of killing, featuring trend lines and custom colors for the T cell products tested. Similarly, line graphs were used to visualize 3-day IVs values across the rounds for the different T cell products. Bar graphs visualize the IVs with customized aesthetics for clarity. Schematic illustrations were created using BioRender.com.

### Additional resources

Clinical trial identifier: NCT04099797, ClinicalTrials.gov/study/NCT04099797. The supplementary materials (Extended Data Figures S1–S11 and Extended Data Tables S1–S4) are provided in a separate file.
