# Supplementary material for: Multi-parametric profiling of IL-7-augmented GD2.CART products in a phase 1 clinical trial
Source: iScience. 2025 Oct 6;28(11):113680. doi: 10.1016/j.isci.2025.113680 (PMC12589891; doi:10.1016/j.isci.2025.113680)
Supplement: Document S1. Figures S1–S11 and Tables S1–S4 [file mmc1.pdf]

iScience, Volume *28*

Supplemental information

Multi-parametric profiling of IL-7-augmented GD2.CART products in a phase 1 clinical trial

Sarah Schulenberg, Martí Farrera-Sal, Michelle Loeser, Lukas Ehlen, Stephan Schlickeiser, Samira Picht, Lena Peter, Marco Mai, Candise L. Tat, Jacqueline Keye, Desiree Kunkel, Frank Lin, Cliona M. Rooney, Bilal Omer, and Michael Schmueck-Henneresse

Extended Data Figures

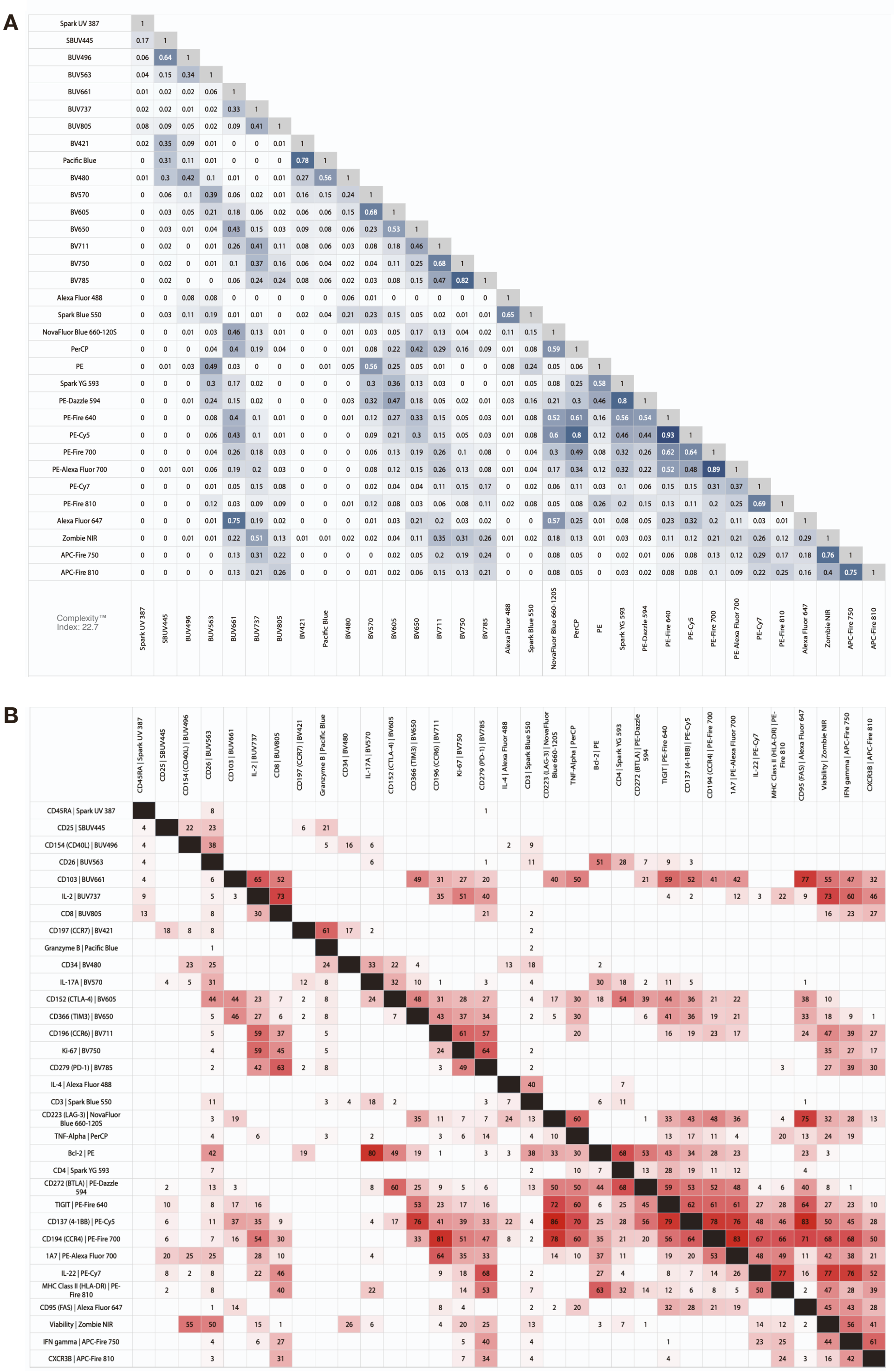

Figure S1 – Similarity Index Matrix and Stain Index Reduction Matrix of the 33-color FSFC panel. (**A**) The Similarity Index Matrix is shown with a Complexity Index of 22.7 for the 33 fluorochromes in the FSFC panel, where low values are in white and high values in dark blue. (**B**) The Stain Index Reduction Matrix for the FSFC panel shows the percentage reduction in the stain index of a fluorochrome (column) due to another fluorochrome's signal (row), with low values in white and high values in red. The tables were assessed using the Cytek Cloud Panel Builder.

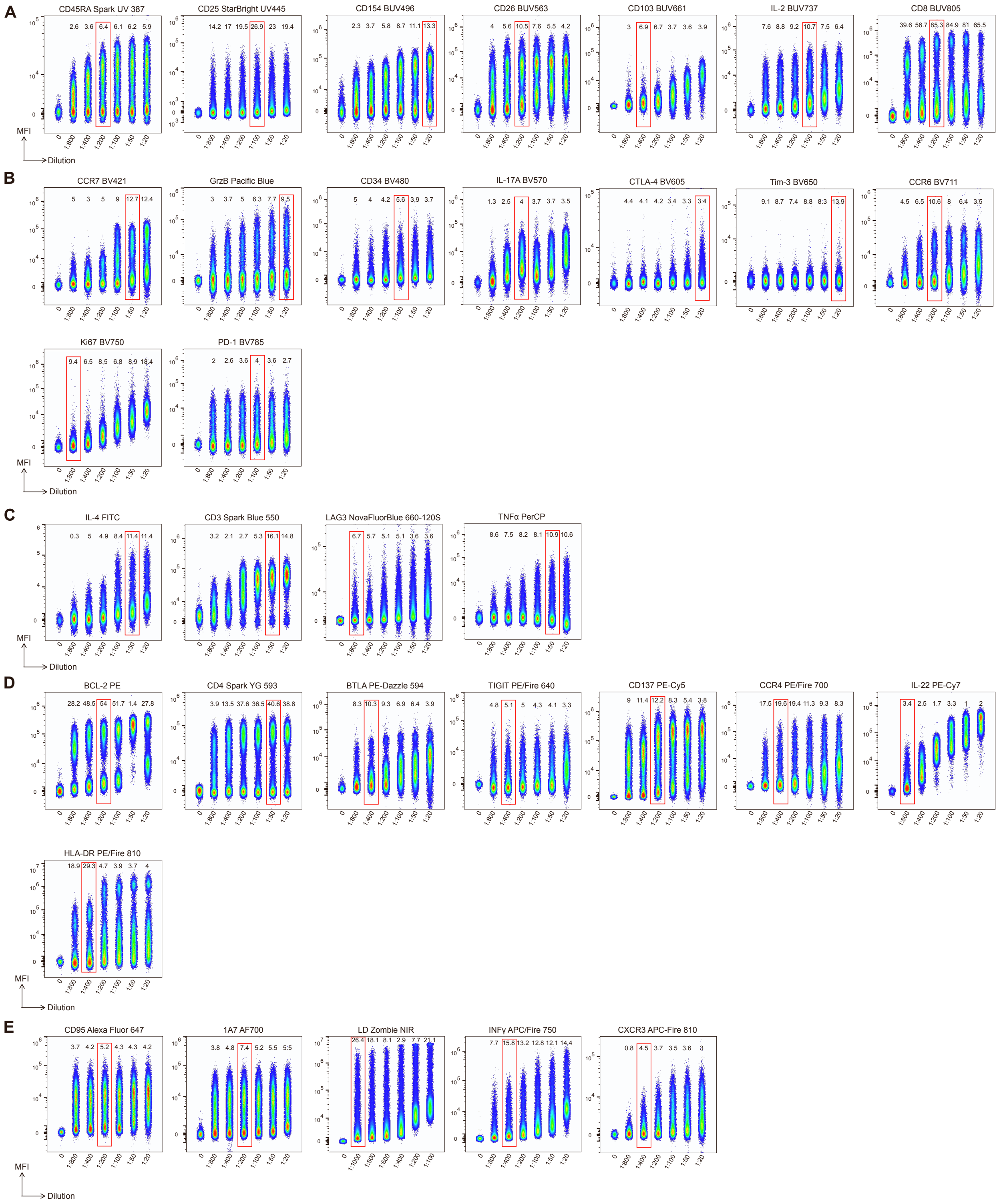

A
CD45RA Spark UV 387
2.6 3.6 6.4 6.1 6.2 5.9
CD25 StarBright UV445
14.2 17 19.5 26.9 23 19.4
CD154 BUV496
2.3 3.7 5.8 8.7 11.1 13.3
CD26 BUV563
4 5.6 10.5 7.6 5.5 4.2
CD103 BUV661
3 6.9 6.7 3.7 3.6 3.9
IL-2 BUV737
7.6 8.8 9.2 10.7 7.5 6.4
CD8 BUV805
39.6 56.7 85.3 84.9 81 65.5
MFI
Dilution
B
CCR7 BV421
5 3 5 9 12.7 12.4
GrzB Pacific Blue
3 3.7 5 6.3 7.7 9.5
CD34 BV480
5 4 4.2 5.6 3.9 3.7
IL-17A BV570
1.3 2.5 4 3.7 3.7 3.5
CTLA-4 BV605
4.4 4.1 4.2 3.4 3.3 3.4
Tim-3 BV650
9.1 8.7 7.4 8.8 8.3 13.9
CCR6 BV711
4.5 6.5 10.6 8 6.4 3.5
Ki67 BV750
9.4 6.5 8.5 6.8 8.9 18.4
PD-1 BV785
2 2.6 3.6 4 3.6 2.7
MFI
Dilution
C
IL-4 FITC
0.3 5 4.9 8.4 11.4 11.4
CD3 Spark Blue 550
3.2 2.1 2.7 5.3 16.1 14.8
LAG3 NovaFluorBlue 660-120S
6.7 5.7 5.1 5.1 3.6 3.6
TNFα PerCP
8.6 7.5 8.2 8.1 10.9 10.6
MFI
Dilution
D
BCL-2 PE
28.2 48.5 54 51.7 1.4 27.8
CD4 Spark YG 593
3.9 13.5 37.6 36.5 40.6 38.8
BTLA PE-Dazzle 594
8.3 10.3 9.3 6.9 6.4 3.9
TIGIT PE/Fire 640
4.8 5.1 5 4.3 4.1 3.3
CD137 PE-Cy5
9 11.4 12.2 8.3 5.4 3.8
CCR4 PE/Fire 700
17.5 19.6 19.4 11.3 9.3 8.3
IL-22 PE-Cy7
3.4 2.5 1.7 3.3 1 2
HLA-DR PE/Fire 810
18.9 29.3 4.7 3.9 3.7 4
MFI
Dilution
E
CD95 Alexa Fluor 647
3.7 4.2 5.2 4.3 4.3 4.2
1A7 AF700
3.8 4.8 7.4 5.2 5.5 5.5
LD Zombie NIR
26.4 18.1 8.1 2.9 7.7 21.1
INFγ APC/Fire 750
7.7 15.8 13.2 12.8 12.1 14.4
CXCR3 APC-Fire 810
0.8 4.5 3.7 3.5 3.6 3
MFI
Dilution

Figure S2 – Titrations of fluorochrome-coupled antibodies and dyes used in the FSFC panel. For the titration, each antibody was diluted to the final concentrations of 0 (unstained control), 1:800, 1:400, 1:200, 1:200, 1:50, and 1:20. The viablility dye Zombie NIR was diluted to 1:1000, 1:800, 1:600, 1:400, 1:200, and 1:100. Antibody titrations for CD45RA, CD103, CD8, CCR7, CCR6, CD3, CD4, BTLA, TIGIT, CCR4, CD95 and CXCR3 were performed on un-activated PBMCs, for CD25, CD154, CD26, IL-2, GrzB, IL-17A, CTLA-4, Tim-3, Ki67, PD-1, IL-4, LAG-3, TNFα, BCL-2, CD137, IL-22, HLA-DR, and IFNγ on activated PBMCs, and for CD34 and 14G2a (1A7) on C7R-GD2.CART cells. Stained samples were recorded at a full spectrum cytometer (Cytek Aurora). Files were concatenated for visualization of positive and negative populations across dilutions. Final concentrations (red squares) were selected based on the stain index (SI) stated and the absence of shifts in the negative population due to nonspecific binding. The SI was calculated using the formula: SI = (MFI_positive - MFI_negative) / (2 * SD_negative), where MFI represents the mean fluorescence intensity and SD is the standard deviation of the positive or negative population. Titrations are shown for the excitation by (**A**) UV laser (355 nm), (**B**) violet laser (405 nm), (**C**) blue laser (488 nm), (**D**) yellow-green laser (561 nm) and (**E**) red laser (640 nm).

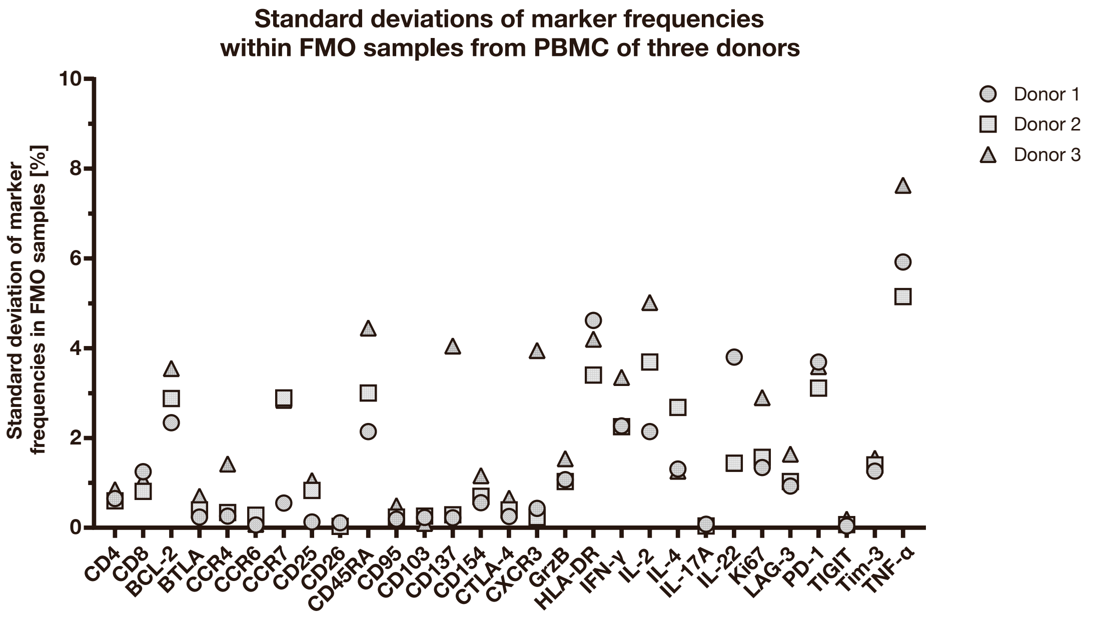

Figure S3 – Marker frequencies within fluorescence minus one (FMO) samples. FMO samples were generated from cultured peripheral blood mononuclear cells (PBMCs) from three different healthy donors (n = 3). For this, PBMCs were polyclonally activated for 48 h, cultured for additional 14 days, and restimulated with PMA and ionomycin 16 h before staining. FMO staining of CD45RA, CD25, CD26, CD103, CD8, CCR7, CD34, and CTLA-4 was stained on the T cells of donor 1, for Tim-3, CCR6, PD-1, CD4, BTLA, TIGIT, CD137, CCR4, HLA-DR, CD95 and 14G2a (1A7) on those of donor 2 and the FMOs for the marker CXCR3, CD154, IL-2, GrzB, IL-17A, Ki67, IL-4, LAG-3, BCL-2, IL-22, and IFN-γ were stained on the T cells of donor 3. Prior gating was performed on $CD3^+$ alive cells. Standard deviations of the frequencies of each marker within the individual donors, and hence, the certain FMO samples are shown. Donor 1 (n = 9), donor 2 (n = 13), donor 3 (n = 13). Image was created with GraphPad Prism.

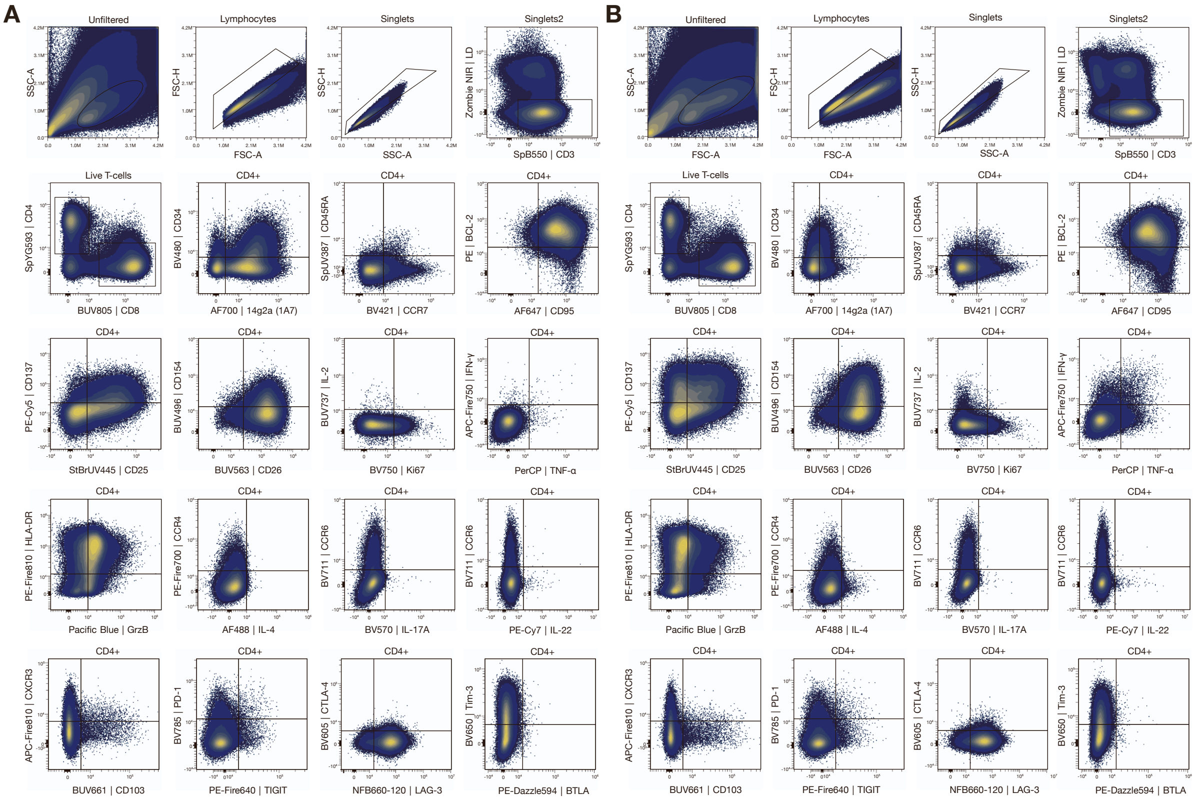

Figure S4 – Marker expression in CD4⁺ T cells from clinical products. T cell products harboring the GD2-CAR alone (n = 3) or in combination with the C7R (n = 7) were stimulated with GD2-expressing LAN-1 tumor cells at a 1:1 ratio for 12 hours. Samples were stained with a 33-color panel to detect T cell markers using full spectrum flow cytometry. In (**A**) unstimulated and (**B**) stimulated T cell products, single events were identified based on size and granularity (FSC, SSC), followed by doublet exclusion. Viable T cells were identified by excluding Zombie-dye-positive events and including $CD3^+$ events. $CD4^+CD8^-$ T cells are shown, with the expression of markers relevant for memory differentiation, activation, apoptosis regulation, proliferation, cytokine production, cytotoxicity, tissue infiltration, and exhaustion-related checkpoint expression displayed as an overlay of the T cell products analyzed (n = 10).

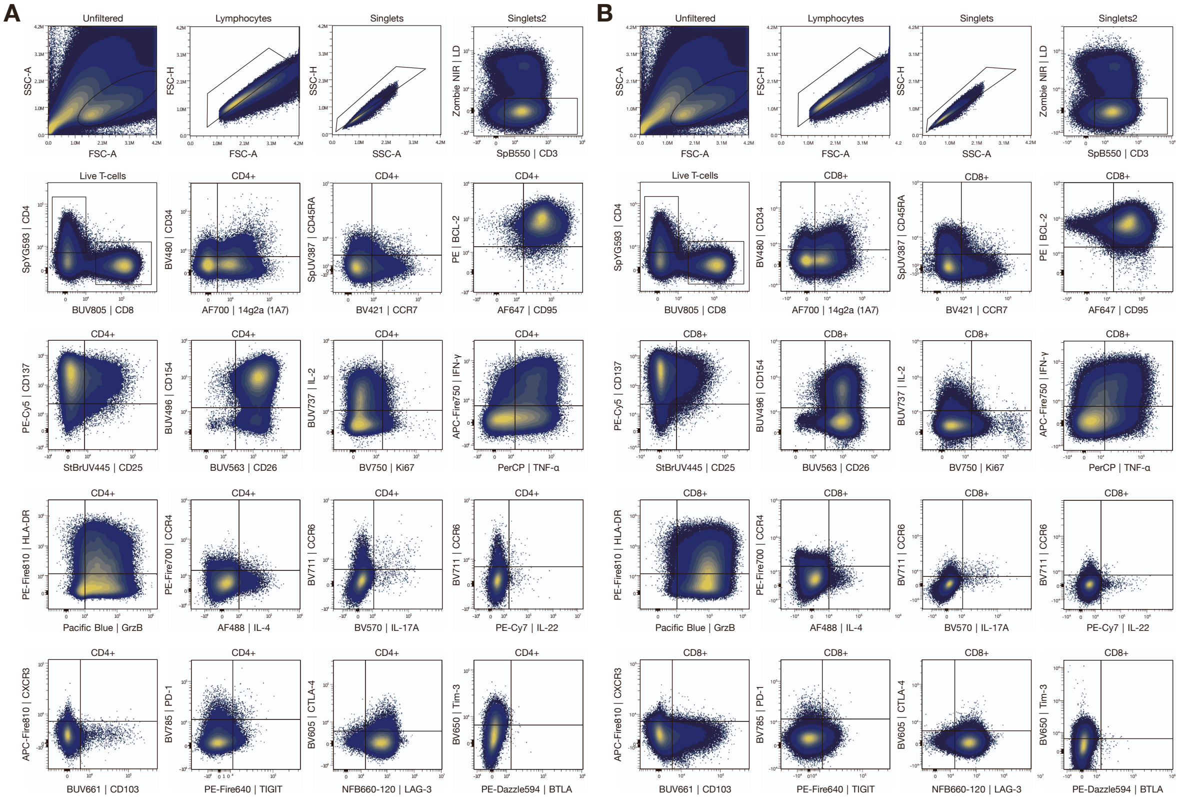

Figure S5 – Marker expression in T cell products following PMA and Ionomycin stimulation. T cell products harboring the GD2-CAR alone (n = 3) or in combination with the C7R (n = 7) were stimulated with PMA and ionomycin for 16 hours. Samples were stained with a 33-color panel to detect T cell markers using full spectrum flow cytometry. In stimulated T cell products, single events were identified based on size and granularity (FSC, SSC), followed by doublet exclusion. Viable T cells were identified by excluding Zombie-dye-positive events and including $CD3^+$ events. (**A**) $CD4^+$CD8- T cells and (**B**) $CD4^-CD8^+$ T cells are shown, with the expression of markers relevant for memory differentiation, activation, apoptosis regulation, proliferation, cytokine production, cytotoxicity, tissue infiltration, and exhaustion-related checkpoint expression displayed as an overlay of the T cell products analyzed (n = 10).

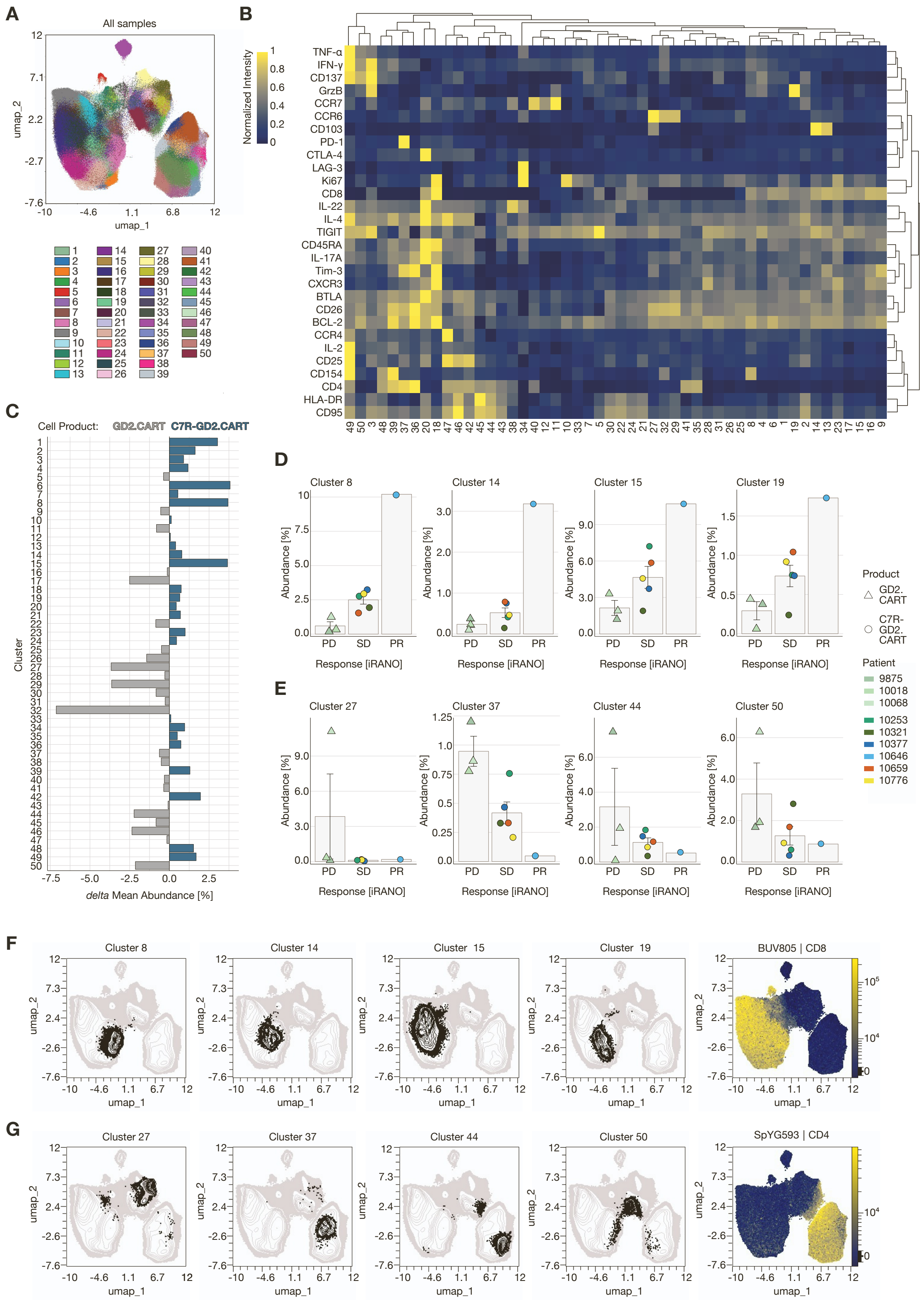
A
All samples
B
Normalized Intensity
C
Cell Product: GD2.CART C7R-GD2.CART
Cluster
delta Mean Abundance [%]
D
Cluster 8
Cluster 14
Cluster 15
Cluster 19
Abundance [%]
Response [iRANO]
Product
GD2. CART
C7R- GD2. CART
Patient
9875
10018
10068
10253
10321
10377
10646
10659
10776
E
Cluster 27
Cluster 37
Cluster 44
Cluster 50
F
BUV805 | CD8
G
SpYG593 | CD4
umap_1
umap_2
TNF-α
IFN-γ
CD137
GrzB
CCR7
CCR6
CD103
PD-1
CTLA-4
LAG-3
Ki67
CD8
IL-22
IL-4
TIGIT
CD45RA
IL-17A
Tim-3
CXCR3
BTLA
CD26
BCL-2
CCR4
IL-2
CD25
CD154
CD4
HLA-DR
CD95

Figure S6 – Detailed FlowSOM cluster analysis supporting the metaclustering approach in Fig. 3. (**A**) FlowSOM clustering (k = 50) performed with max equal 100,000 $CD3^+$ alive cells from each T-cell product (n = 10) in LAN-1-stimulated and unstimulated condition (total n = 20) including all markers except CD3 and the viability dye used for pre-filtering and markers detecting the CAR and C7R (14g2a and CD34, respectively), prior to consolidation into the 35 metaclusters. (**B**) Composition heatmap with hierarchical clustering (average) shows protein abundance per cluster (min-max-scaling). (**C**) Delta abundance comparison between GD2.CART-only and C7R-GD2.CART products, calculated by subtracting the mean abundance of each cluster in the C7R-GD2.CAR group (n = 6) from the GD2.CART group (n = 3). (**D, E**) Detailed view of the eight individual clusters that contribute to the metaclusters associated with clinical outcomes: (**D**) four clusters positively associated with clinical response and (**E**) four clusters negatively associated with clinical response, with mean abundance (%) shown alongside corresponding iRANO scores for patients with progressive disease (PD), stable disease (SD), or partial response (PR). Data are represented as mean +/- SEM. (**F, G**) Cells of the clusters are displayed in the UMAP (black), associating their location with CD4 and CD8 expressing cells represented by the fluorescence intensity of the respective antibodies (color continuous scale).

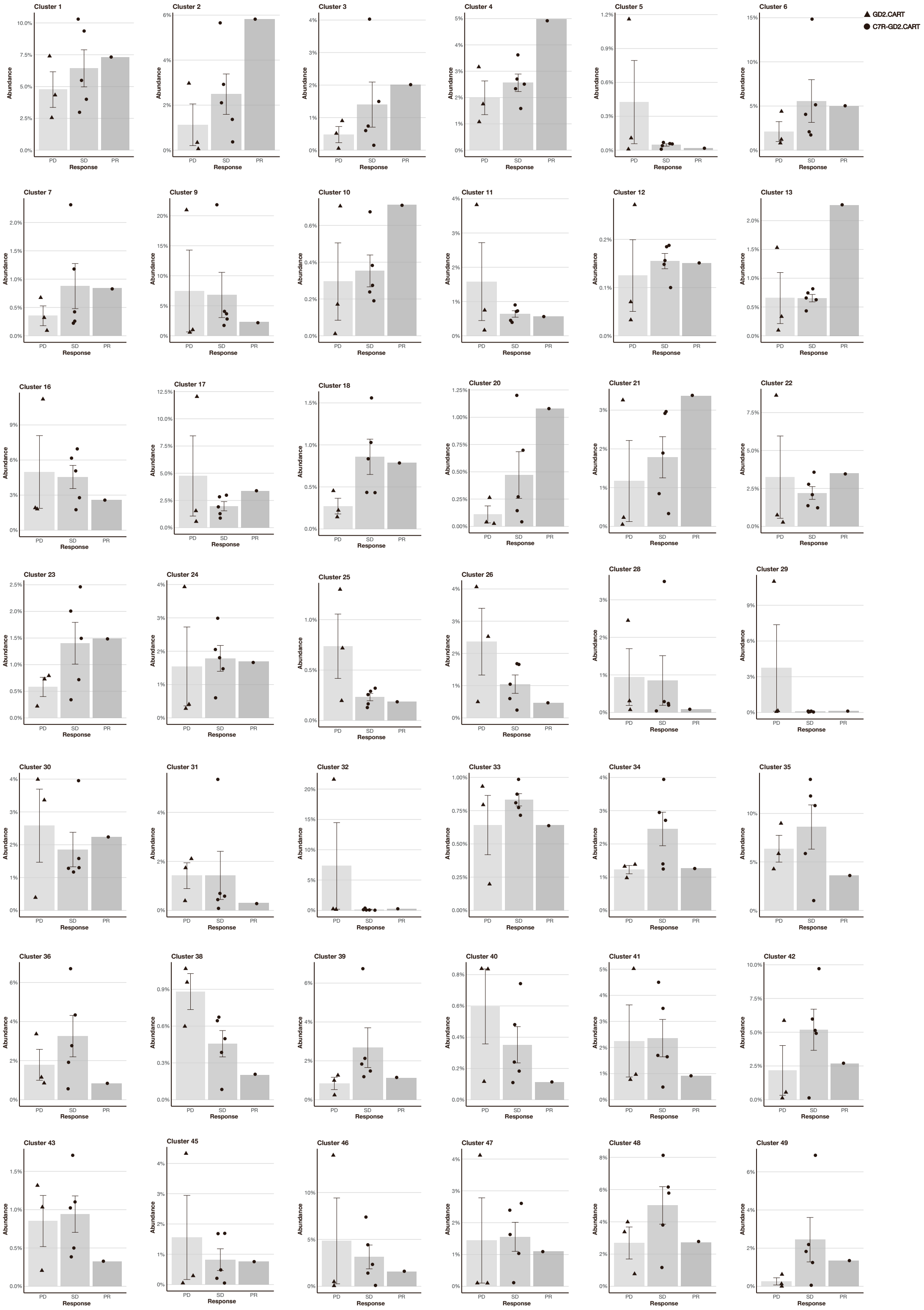
GD2.CART
C7R-GD2.CART
Cluster 1
Cluster 2
Cluster 3
Cluster 4
Cluster 5
Cluster 6
Cluster 7
Cluster 9
Cluster 10
Cluster 11
Cluster 12
Cluster 13
Cluster 16
Cluster 17
Cluster 18
Cluster 20
Cluster 21
Cluster 22
Cluster 23
Cluster 24
Cluster 25
Cluster 26
Cluster 28
Cluster 29
Cluster 30
Cluster 31
Cluster 32
Cluster 33
Cluster 34
Cluster 35
Cluster 36
Cluster 38
Cluster 39
Cluster 40
Cluster 41
Cluster 42
Cluster 43
Cluster 45
Cluster 46
Cluster 47
Cluster 48
Cluster 49
Abundance
Response
PD
SD
PR

Figure S7 – Mean FlowSOM cluster abundance and corresponding iRANO classifications for T cell products. FlowSOM clustering (k = 50) performed with max equal 100,000 CD3+ alive cells from each T cell product (n = 10) in LAN-1-stimulated and unstimulated condition (total n = 20) including all markers except CD3 and the viability dye used for pre-filtering and markers detecting the CAR and C7R (14G2a (1A7) and CD34, respectively). Mean cluster abundance (%) for each patient is shown alongside the corresponding iRANO scores for T cell products in patients with progressive disease (PD), stable disease (SD), or partial response (PR) after 6 weeks of treatment. The abundance of all clusters is displayed, apart from the eight clusters mentioned in Fig. S6. Data are represented as mean +/- SEM.

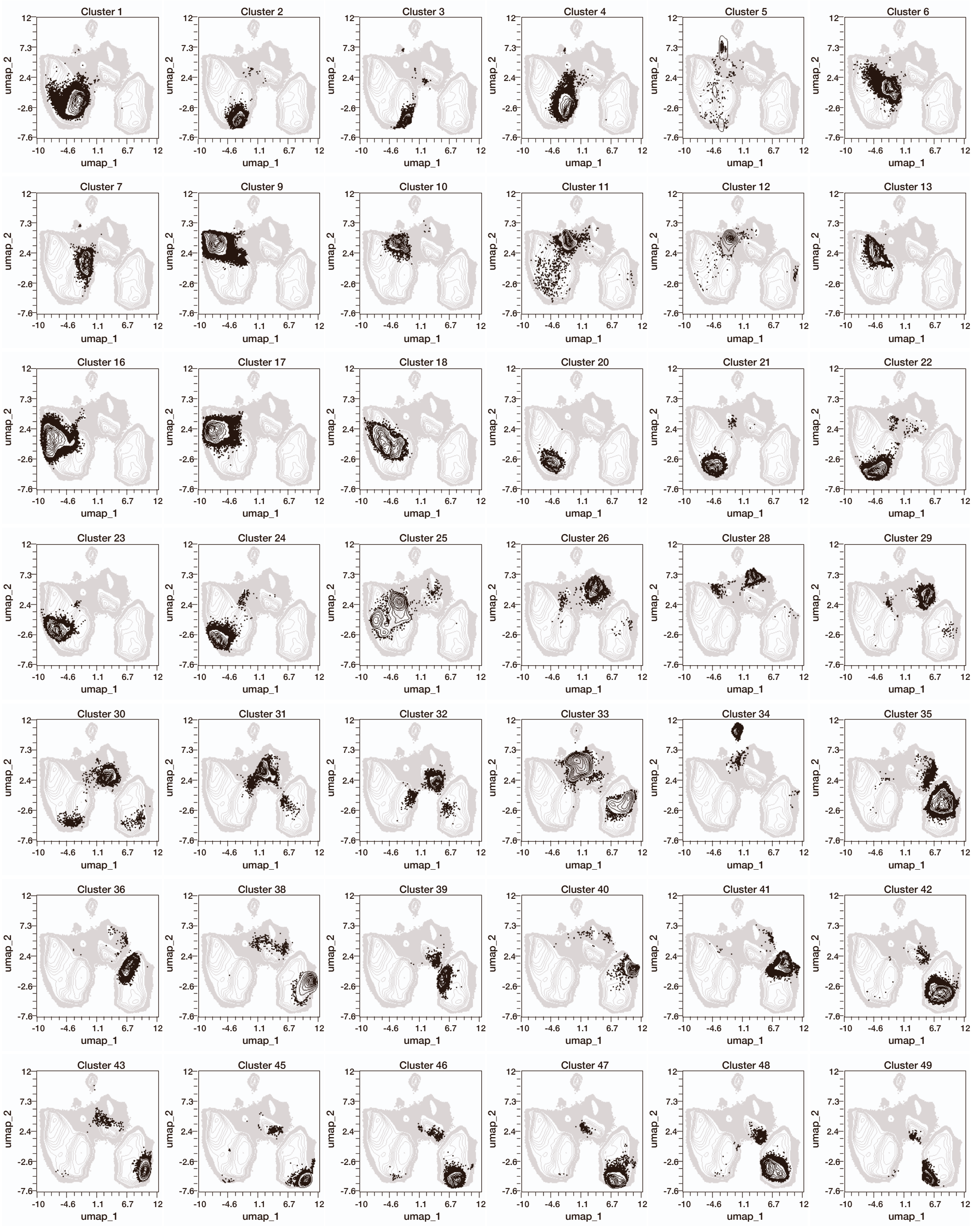

Figure S8 – FlowSOM Cluster Visualization in UMAP of alive T cells from T cell products. FlowSOM clustering (k = 50) performed with max equal 100,000 CD3+ alive cells from each T cell product (n = 10) in LAN-1-stimulated and unstimulated condition (total n = 20) including all markers except CD3 and the viability dye used for pre-filtering and markers detecting the CAR and C7R (14G2a (1A7) and CD34, respectively). Cells of all clusters (black), apart from the eight clusters mentioned in Fig. S6, are displayed in the respective UMAP.

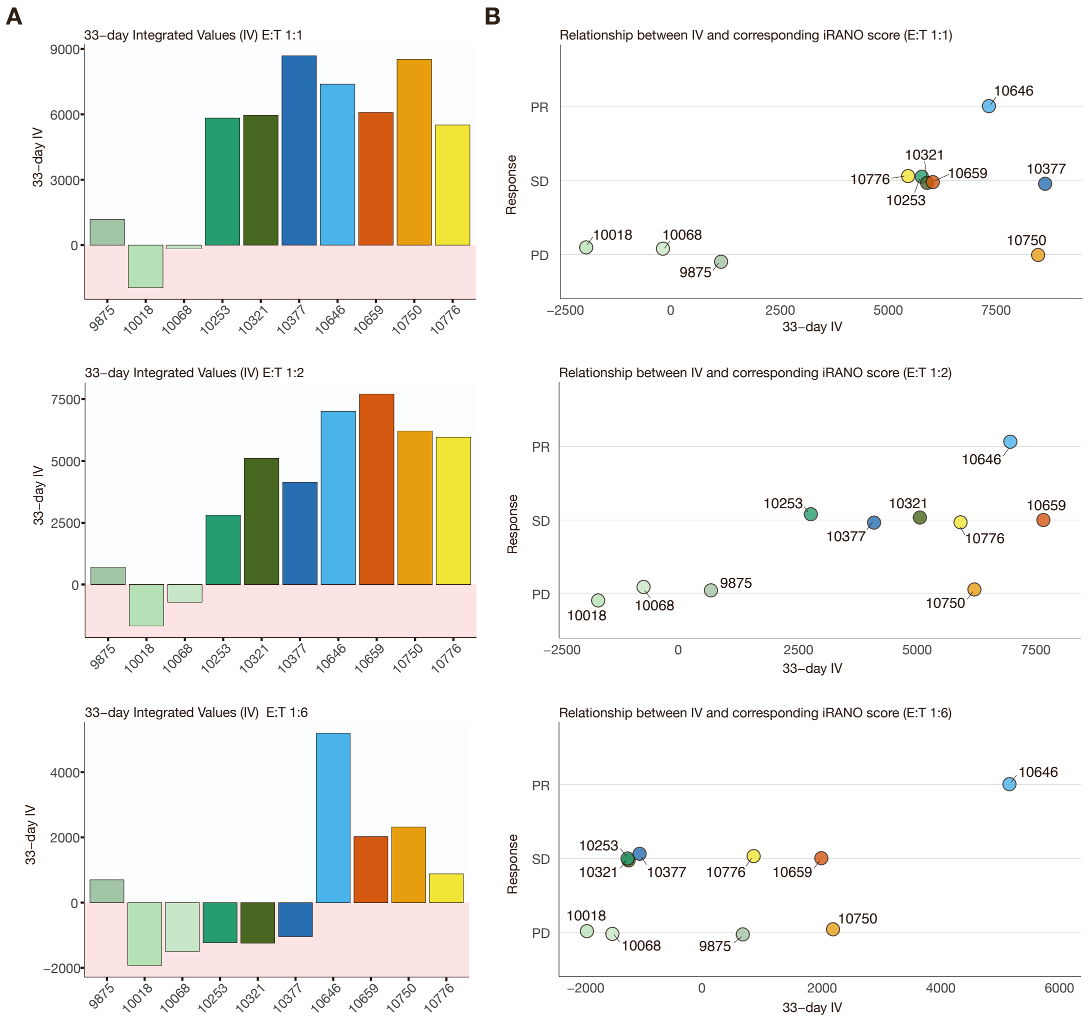

Figure S9 – Serial killing dynamics of GD2.CART cell products at effector-target ratios 1:1, 1:2, and 1:6. T cell products were challenged every 3 days with a GD2-positive LAN-1 tumor cell line expressing GFP for a total of 11 rounds. The serial killing dynamics of each T cell product were evaluated using a 3-day integrated value (IV), calculated by summing the killing percentages of each CART cell product for each round. (**A**) A 33-day IV, derived from the cumulative 3-day IVs of each CART cell product at effector-target ratios of 1:1, 1:2, and 1:6, is presented as bar graphs. (**B**) The association between killing and clinical response is illustrated by comparing the 33-day IV of the T cell products at the specified effector-target ratios with the iRANO scores of the respective patients.

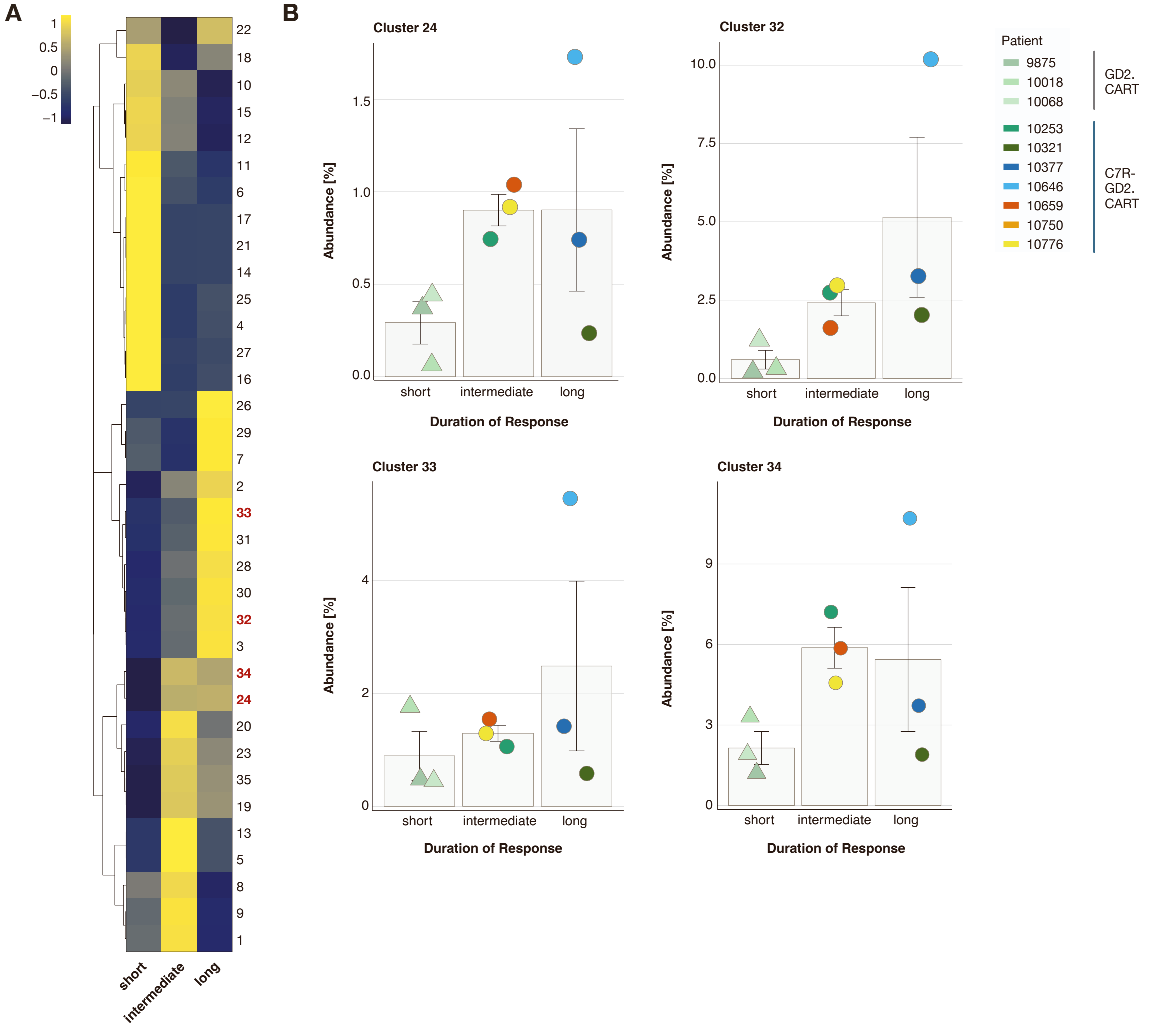

Figure S10 – Treatment duration with GD2.CART cell products. Clinical responses of the autologous CART cell products were categorized based on treatment duration into three groups: short (< 1 month), intermediate (< 5 months), and long (> 5 months). (**A**) A composition heatmap with hierarchical clustering (average) shows protein abundance per cluster (z-score) in the three groups. Cluster that were positively associated with clinical response (PR, see Figure 3) are marked in red. (**B**) The abundance of the four clusters positively associated with response is displayed for the three response duration groups. Data are represented as mean +/- SEM.

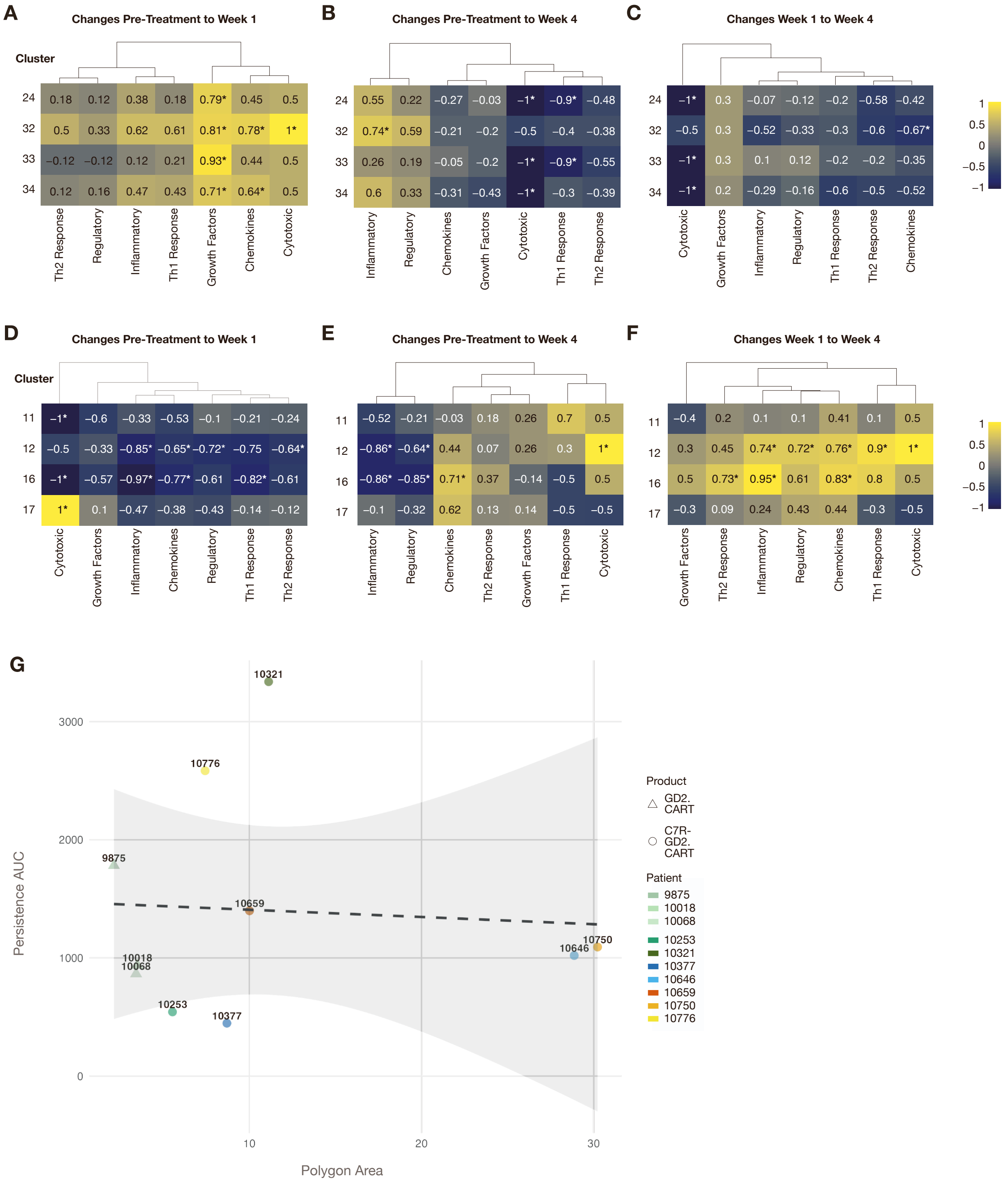
A
Changes Pre-Treatment to Week 1
Cluster
24 0.18 0.12 0.38 0.18 0.79* 0.45 0.5
32 0.5 0.33 0.62 0.61 0.81* 0.78* 1*
33 −0.12 −0.12 0.12 0.21 0.93* 0.44 0.5
34 0.12 0.16 0.47 0.43 0.71* 0.64* 0.5
Th2 Response
Regulatory
Inflammatory
Th1 Response
Growth Factors
Chemokines
Cytotoxic
B
Changes Pre-Treatment to Week 4
24 0.55 0.22 −0.27 −0.03 −1* −0.9* −0.48
32 0.74* 0.59 −0.21 −0.2 −0.5 −0.4 −0.38
33 0.26 0.19 −0.05 −0.2 −1* −0.9* −0.55
34 0.6 0.33 −0.31 −0.43 −1* −0.3 −0.39
Inflammatory
Regulatory
Chemokines
Growth Factors
Cytotoxic
Th1 Response
Th2 Response
C
Changes Week 1 to Week 4
24 −1* 0.3 −0.07 −0.12 −0.2 −0.58 −0.42
32 −0.5 0.3 −0.52 −0.33 −0.3 −0.6 −0.67*
33 −1* 0.3 0.1 0.12 −0.2 −0.2 −0.35
34 −1* 0.2 −0.29 −0.16 −0.6 −0.5 −0.52
Cytotoxic
Growth Factors
Inflammatory
Regulatory
Th1 Response
Th2 Response
Chemokines
1
0.5
0
−0.5
−1
D
Changes Pre-Treatment to Week 1
Cluster
11 −1* −0.6 −0.33 −0.53 −0.1 −0.21 −0.24
12 −0.5 −0.33 −0.85* −0.65* −0.72* −0.75 −0.64*
16 −1* −0.57 −0.97* −0.77* −0.61 −0.82* −0.61
17 1* 0.1 −0.47 −0.38 −0.43 −0.14 −0.12
Cytotoxic
Growth Factors
Inflammatory
Chemokines
Regulatory
Th1 Response
Th2 Response
E
Changes Pre-Treatment to Week 4
11 −0.52 −0.21 −0.03 0.18 0.26 0.7 0.5
12 −0.86* −0.64* 0.44 0.07 0.26 0.3 1*
16 −0.86* −0.85* 0.71* 0.37 −0.14 −0.5 0.5
17 −0.1 −0.32 0.62 0.13 0.14 −0.5 −0.5
Inflammatory
Regulatory
Chemokines
Th2 Response
Growth Factors
Th1 Response
Cytotoxic
F
Changes Week 1 to Week 4
11 −0.4 0.2 0.1 0.1 0.41 0.1 0.5
12 0.3 0.45 0.74* 0.72* 0.76* 0.9* 1*
16 0.5 0.73* 0.95* 0.61 0.83* 0.8 0.5
17 −0.3 0.09 0.24 0.43 0.44 −0.3 −0.5
Growth Factors
Th2 Response
Inflammatory
Regulatory
Chemokines
Th1 Response
Cytotoxic
1
0.5
0
−0.5
−1
G
Persistence AUC
3000
2000
1000
0
10321
10776
9875
10659
10018
10068
10253
10377
10646
10750
10
20
30
Polygon Area
Product
GD2.CART
C7R-GD2.CART
Patient
9875
10018
10068
10253
10321
10377
10646
10659
10750
10776

Figure S11 – Correlation of immune cell cluster abundance with functional cytokines and cell product persistence. Spearman correlation coefficients illustrate the relationship between the abundance of (**A-C**) four positive-response-associated immune cell clusters (24, 32, 33, 34) and (**D-F**) four negative-response-associated immune cell clusters (11, 12, 16, 17) with delta values of functional cytokine scores measured in peripheral blood. Functional grouping of cytokine levels was performed into biologically relevant categories: Inflammatory Cytokines (IL-1β, IL-6, TNF-α), Th1 Response (IFN-γ, IL-12p70), Th2 Response (IL-4, IL-13, IL-33), Regulatory Cytokines (IL-10, IL-1ra), Chemokines (MCP-1, MIP-1β, IP-10, IL-8), Cytotoxic Factors (Granzyme B, IFN-γ), and Growth Factors (GM-CSF, IL-15, IL-2). Displayed are the changes between (**A, D**) pre-treatment and week 1, (**B, E**) pre-treatment and week 4, and (**C, F**) week 1 and week 4. Positive correlations (yellow) indicate increased cytokine levels associated with higher cluster abundance, while negative correlations (blue) indicate decreased levels. Numbers within the cells represent the correlation coefficients (* = $p < 0.05$). N=10 patients. (**G**) Scatter plot showing the relationship between radar chart polygon area (see Fig. 5) and CAR transgene persistence AUC measured by qPCR over 4 weeks post-infusion. Each point represents an individual patient, with triangles indicating CAR-only products (n=3) and circles indicating CAR.C7R products (n=7). Patient IDs are labeled next to each data point. Persistence AUC was calculated using trapezoidal integration of qPCR measurements at 3 hours, 1 week, 2 weeks, and 4 weeks post-infusion. Gray dashed line shows linear regression fit with 95% confidence interval. Pearson correlation coefficient r = -0.07.

Extended Data Tables

Table S1 – Description of the FSFC markers. For every marker in the FSFC panel, the protein name, the function, and the specific purpose of the marker in the immunophenotyping of T cells is depicted. If not other indicated, the information was obtained from UniProtKB and the entry identifiers reported, otherwise the literature is given.

Marker	Name	Function	Purpose	Source
**14G2a (1A7)**	Anti-14G2a	Idiotype of an anti-GD2 antibody, representing GD2	Identification of GD2.CAR T cells	°
**BCL-2**	Apoptosis regulator BCL-2	Inhibits apoptosis of the cell	Activation state of T cells	P10415
**BTLA**	B- and T lymphocyte attenuator	Inhibitory receptor of T cells	Exhaustion state of T cells	Q7Z6A9
**CCR4**	C-C chemokine receptor type 4	Chemokine receptor for CCL17 and CCL22 mediating chemotaxis	Identification of T cell subsets ($T_{H/C2}$)	P51679
**CCR6**	CC-chemokine receptor type 6	Chemokine receptor for CCL20 mediating chemotaxis and migration	Identification of T cell subsets ($T_{H/C17}$, $T_{H/C22}$)	P51684
**CCR7**	C-C chemokine receptor type 7	Chemokine receptor for CC-chemokine ligand 19 (CCL19) and CCL21 mediating homing of T cells to lymph nodes	Identification of naive, and central memory T cells (T_{CM})	P32248
**CD103 (ITGAE)**	Cluster of differentiation 103 (Integrin alpha-E)	E-cadherin receptor mediating adhesion of T cells to epithelial cells	Assessment of tissue-infiltration capacity of T cells	P38570
**CD137 (TNFRSF 9)**	Cluster of differentiation 137 (Tumor necrosis factor receptor superfamily member 9)	Transmembrane protein acting in costimulation of survival, cytotoxicity, and effector functions of T cells	Activation state of T cells	Q07011
**CD154 (CD40L)**	Cluster of differentiation 154 (CD40 Ligand)	Transmembrane protein acting in costimulation of T cell proliferation and cytokine production	Activation state of T cells	P29965
**CD25 (IL2RA)**	Cluster of differentiation 25 (Interleukin-2 receptor subunit alpha)	Receptor for IL-2	Activation state of T cells	P01589
**CD26 (DPP4)**	Cluster of differentiation 26 (Dipeptidyl peptidase 4)	Glycoprotein membrane receptor acting in costimulation of TCR-mediated T cell activation	Activation state of T cells	P27487
**CD3**	Cluster of differentiation 3	Part of the T cell receptor (TCR)	Identification of T cells	P09693

**CD34**	Hematopoietic progenitor cell antigen CD34	Ectodomain included on C7R Coreceptor design of C7R-GD2.CART cells	Identification of C7R-GD2.CART cells	°°
**CD4**	Cluster of differentiation 4	Coreceptor for MHC class II molecules on T cells	Identification of CD4+ T cells (T helper cells (T_H))	P01730
**CD45RA**	Cluster of differentiation 45 (isoform RA)	Tyrosine phosphatase required for T cell activation via the TCR	Identification of T cell memory subsets	P08575-8
**CD8**	Cluster of differentiation 8	Coreceptor for MHC class I molecules on T cells	Identification of CD8+ T cells (cytotoxic T cells (T_C))	P01732 (CD8A)
**CD95 (FAS)**	Tumor necrosis factor receptor superfamily member 6	Apoptosis-inducing receptor	Apoptosis state of T cells	P25445
**CTLA-4**	Cytotoxic T-lymphocyte protein 4	Inhibitory receptor of T cells	Checkpoint on T cells	P16410
**CXCR3**	C-X-C chemokine receptor type 3	Chemokine receptor for CXCl9, CXCL10, and CXCL11, acting in chemotaxis	Migratory capacity of T cells	P49682
**GrzB**	Granzyme B	Protease in the cytotoxic granules of cytotoxic T and NK cells	Cytotoxic state of T cells	P10144
**HLA-DR**	HLA class II histocompatibility antigen, DR	Part of the MHC class II molecule in antigen-presenting cells, Expressed on activated T cells	Activation state of T cells	P01903
**IFNγ**	Interferon gamma	Type II interferon with anti-viral, -microbial and anti-tumor activity	Activation state of T cells	P01579
**IL-17A**	Interleukin 17A	Effector cytokine in anti-bacterial and fungal immunity	Identification of T cell subsets (T_{H17}, T_{C17}) and activation state	Q16552
**IL-2**	Interleukin 2	Cytokine acting in immune response and tolerance, acting as T cell growth factor, promoting proliferation and differentiation of T cells	Activation state of T cells, T helper cell subtype differentiation	P60568
**IL-22**	Interleukin 22	Cytokine mediating cell survival and proliferation	Identification of T cell subsets ($T_{H/C22}$)	Q9GZX6
**IL-4**	Interleukin 4	Cytokine mediating hematopoiesis, inflammation, and effector T cell response	Activation state of T cells	P05112

**Ki67**	Proliferation marker protein	Maintenance of mitotic chromosomes and chromatin organization	Marker for proliferation of T cells	P46013
**LAG-3**	Lymphocyte activation gene 3 protein	Inhibitory receptor on activated T cells	Checkpoint on T cells	P18627
**PD-1**	Programmed cell death protein 1	Inhibitory receptor of activated T cells	Checkpoint on T cells	Q15116
**TIGIT**	T-cell immunoreceptor with Ig and ITIM domains	Inhibitory receptor of T cells	Checkpoint on T cells	Q495A1
**TIM-3 (HAVCR2)**	T cell immunoglobulin and mucin-domain containing-3	Co-inhibitory receptor of IFNγ-producing T cells	Checkpoint on T cells	Q8TDQ0
**TNFα**	Tumor necrosis factor alpha	Cytokine inducing cell death	Activation state of T cells	P01375

° Sen, G., Chakraborty, M., Foon, K.A., Reisfeld, R.A., and Bhattacharya-Chatterjee, M. (1997). Preclinical evaluation in nonhuman primates of murine monoclonal anti-idiotype antibody that mimics the disialoganglioside GD2. Clin. cancer Res. : Off. J. Am. Assoc. Cancer Res. *3*, 1969–1976.

°° Shum, T., Omer, B., Tashiro, H., Kruse, R.L., Wagner, D.L., Parikh, K., Yi, Z., Sauer, T., Liu, D., Parihar, R., et al. (2017). Constitutive Signaling from an Engineered IL7 Receptor Promotes Durable Tumor Elimination by Tumor-Redirected T Cells. Cancer Discov. *7*, 1238–1247. https://doi.org/10.1158/2159-8290.cd-17-0538.

Table S2 – Reference controls (CTRL) for spectral unmixing. For every marker in the FSFC panel, a fluorescence spectrum reference was provided on beads or cells.

Marker	Fluorochrome	Reference CTRL
**CD45RA**	Spark UV387	Beads
**CD25**	StarBright UV445	Cells
**CD154**	BUV496	Cells
**CD26**	BUV563	Cells
**CD103**	BUV661	Beads
**IL-2**	BUV737	Cells
**CD8**	BUV805	Cells
**CCR7**	BV421	Cells
**GrzB**	PacBlue	Cells
**CD34**	BV480	Beads
**IL-17A**	BV570	Cells
**CTLA-4**	BV605	Cells
**TIM-3**	BV650	Cells
**CCR6**	BV711	Cells
**KI67**	BV750	Cells
**PD-1**	BV785	Beads
**IL-4**	AF488	Cells
**CD3**	Spark Blue 550	Cells
**LAG-3**	Nova Fluor Blue 660-120S	Cells
**TNFα**	PerCP	Beads
**BCL-2**	PE	Cells
**CD4**	Spark YG 593	Cells
**BTLA**	PE-Dazzle	Beads
**TIGIT**	PE-Fire640	Beads
**CD137**	PE-Cy5	Beads
**CCR4**	PE-Fire700	Beads
**IL-22**	PE-Cy7	Cells
**HLA-DR**	PE-Fire810	Cells
**CD95**	AF647	Beads
**14G2a (1A7)**	AF700	Cells
**Zombie**	NIR	Cells
**IFNγ**	APC-Fire750	Cells
**CXCR3**	APC-Fire810	Cells

Table S3 – CAR-expression in T cell products. For every T cell product, the frequency of $CD3^+$ alive cells expressing the GD2.CAR (CAR^+) and the CAR together with the C7R ($CAR^+/C7R^+$) is given, determined on unstimulated cells after thawing.

Product	CAR^+	$CAR^+/C7R^+$
**#9875**	89.7	0.2
**#10018**	23.8	0.0
**#10068**	79.5	0.2
**#10253**	56.0	16.4
**#10321**	57.2	12.7
**#10377**	31.6	43.5
**#10646**	54.4	26.7
**#10659**	49.3	25.8
**#10750**	70.8	10.4
**#10776**	54.8	26.0

Table S4 – Response and duration of response in patients receiving T cell products. For every T cell product, the clinical response (iRANO score) of the respective patient after 6 weeks and the corresponding duration of response is given.

Product	Response (6 weeks)	Duration of Response	
**#9875**	PD	3 weeks	short
**#10068**	PD	2 weeks	short
**#10018**	PD	2-3 weeks	short
**#10253**	SD	2 months	intermediate
**#10321**	SD	15 months	long
**#10377**	SD	9 months	long
**#10659**	SD	4 months	intermediate
**#10646**	PR	5 months	long
**#10750**	PD	0 months	-
**#10776**	SD	4 months	intermediate
